# Nitrogen and phosphorus stress as a tool to induce lipid production in microalgae

**DOI:** 10.1186/s12934-023-02244-6

**Published:** 2023-11-20

**Authors:** Yevhen Maltsev, Maxim Kulikovskiy, Svetlana Maltseva

**Affiliations:** https://ror.org/03hg8g849 grid.465284.9 0000 0001 1012 9383 К.А. Timiryazev Institute of Plant Physiology RAS, IPP RAS, Moscow, 127276 Russia

**Keywords:** Algae, Nitrogen, Nutrient media, Phosphorus, Principal component analysis

## Abstract

Microalgae, capable of accumulating large amounts of lipids, are of great value for biodiesel production. The high cost of such production stimulates the search for cultivation conditions that ensure their highest productivity. Reducing the content of nitrogen and phosphorus in the culture medium is widely used to change the content and productivity of lipids in microalgae. Achieving the right balance between maximum growth and maximum lipid content and productivity is the primary goal of many experimental works to ensure cost-effective biodiesel production from microalgae. The content of nitrogen and phosphorus in nutrient media for algal cultivation after converted to nitrogen (-N) and phosphorus (-P) lies in an extensive range: from 0.007 g L^− 1^ to 0.417 g L^− 1^ and from 0.0003 g L^− 1^ to 0.227 g L^− 1^ and N:P ratio from 0.12:1 to 823.33:1. When studying nutritional stress in microalgae, no single approach is used to determine the experimental concentrations of nitrogen and phosphorus. This precludes the possibility of correct interpretation of the data and may lead to erroneous conclusions. This work results from the systematisation of information on using nitrogen and phosphorus restriction to increase the lipid productivity of microalgae of different taxonomic and ecological groups to identify future research directions. The results of 301 experiments were included in the analysis using the principal components method. The investigation considered various divisions and classes: Cyanobacteria, Rhodophyta, Dinophyta, Haptophyta, Cryptophyta, Heterokontophyta/Ochrophyta (Bacillariophyceae, Eustigmatophyceae, Xanthophyceae), Chlorophyta, and also the ratio N:P, the time of the experiment, the light intensity during cultivation. Based on the concentrations of nitrogen and phosphorus existing in various nutrient media, a general scheme for designating the supply of nutrient media for nitrogen (as NO_3_^−^ or NH_4_+, N g L^− 1^) and phosphorus (as РO_4_^−^, P g L^− 1^) has been proposed: replete -N (˃0.4 g L^− 1^), moderate -N (0.4–0.2), moderate N-limitation (0.19–0.1), strong N-limitation (˂0.1), without nitrogen (0), replete -Р (˃0.2), moderate -P (0.2–0.02), moderate P-limitation (0.019–0.01), strong P-limitation (˂0.01), without phosphorus (0).

**Supplementary Information:**

The online version contains supplementary material available at 10.1186/s12934-023-02244-6.

## Introduction

Solving the energy problem is one of the urgent tasks of modernity. Among the promising areas is the use of lipid-rich microalgae biomass for the production of biodiesel, a third-generation fuel [1–10]. Numerous studies have shown that the lipid content in the biomass of some microalgae can reach up to 75% [11]. This value is several times higher than the lipid content (15–25%) in the biomass of soybeans, one of the most used oil crops [1, 12, 13].

Lipids are microalgae’s most important energy source, as in other living organisms. They also form cell membranes and can be precursors or intermediates of cell signalling systems that respond to changing environmental conditions [14]. For the production of biodiesel from microalgae, not only the high content of lipids is essential, but also their composition. The most significant value in this regard is the amount of triacylglycerol (TAG), which is the main energy substrate of cells. Its amount in some microalgae can reach up to 80% of the total amount of lipids [2, 15]. Also of great importance is the composition of fatty acids that take part in the formation of lipid molecules. In the composition of various classes of lipids, as well as in the free state in microalgae cells, 135 fatty acids were noted, differing in the length of the hydrocarbon chain, and the presence and number of double bonds [16]. We know that a high content of polyunsaturated fatty acids (PUFAs) in the biomass of microalgae during the production of biodiesel is undesirable because, during storage, they quickly oxidise and degrade the quality of the fuel [1, 17–19]. On the contrary, a high content of saturated fatty acids leads to an increase in the cetane number, and a sufficient amount of monounsaturated fatty acids significantly improves the fluidity of biodiesel fuel at low temperatures [20]. C16-C18 fatty acids can improve the combustion characteristics of biodiesel [21]. Thus, microalgae with a high content of saturated and monounsaturated fatty acids in lipids are the most promising for biodiesel fuel production.

The content of PUFAs, monounsaturated fatty acids (MUFAs) and saturated fatty acids in various classes of lipids is different. Neutral lipids and TAGs are rich in MUFAs, while polar lipids (glycolipids and phospholipids) show a higher percentage of PUFAs [22, 23]. Therefore, the high content of neutral lipids is often a critical factor in determining the suitability of microalgae for biodiesel.

Despite significant advances in studying microalgae as a feedstock for biodiesel production, the industrial cultivation of microalgae has not yet become widespread. The main constraint is the low profitability of such production [24]. Various strategies are used to overcome this limitation, and several recent reviews are devoted to their discussion [25, 26]. Molecular and metabolic engineering, and gene editing technology (CRISPR/Cas9) are used to induce high lipid levels in addition to nutrition [27–29].

Much attention is paid to optimising microalgae biomass composition and increasing biodiesel production’s profitability by changing the cultivation conditions. It is, for example, the use of abiotic stress factors during cultivation [30, 31], including nutrient restriction. There is evidence of an increase in lipid content and lipid productivity in some microalgae under nitrogen starvation conditions [32, 33]. There is evidence of a significant increase in lipid levels during cultivation with nitrogen restriction in *Tetradesmus obliquus*, *Chlorella vulgaris* and *Mychonastes homosphaera* [34]; in *Auxenochlorella pyrenoidosa* [35] in *Isochrysis galbana* [36]; in *Microcystis* [37]. It was reported that in *Chloroidium ellipsoideum* (CUH/Al/MW-189) and *Chlorococcum infusionum* (CUH/Al/MW-190), the total lipid content was maximum in the absence of nitrogen in the medium, and an increase in nitrate content resulted in a decrease in lipid content [38]. Simultaneously with an increase in lipid content, nitrogen starvation can decrease PUFA production, which is valuable for biodiesel production [1, 17–19]. This effect was observed in *Isochrysis galbana* [39] and *Chlorella sorokiniana* [33]. In addition to nitrogen, limiting the amount of phosphorus in nutrient media is also accompanied by changes in the content and qualitative composition of microalgae lipids [40, 41]. For example, under phosphorus starvation, the content of total lipids increased threefold in *Chloroidium ellipsoideum* (CUH/Al/MW-189), and twofold in *Chlorococcum infusionum* (CUH/Al/MW-190) [42].

Recent studies support the ability to regulate microalgae growth and lipid accumulation through precision nutrient restriction [43]. However, stress conditions caused by a lack of nitrogen or phosphorus can decrease the growth rate of microalgae, biomass accumulation and, accordingly, the reduction in the final lipid yield [44–46]. Therefore, it is necessary to select such concentrations of nitrogen and phosphorus in the cultivation medium that can provide both rapid growth and accumulation of lipids by cells. From a practical point of view, it is essential then to determine the ranges of the optimal and deficient content of nitrogen and phosphorus in the cultivation medium, especially for those species of microalgae, for which the ability to accumulate lipids in large quantities is already known.

Studies over the past few decades have described the growth response, lipid and FA changes of many microalgae species to nutrient restriction. Such observations are of decisive importance for understanding the general patterns of the reaction of lipid-producing microalgae to nitrogen or phosphorus starvation. However, widely differing experimental approaches preclude direct comparisons between studies. Thus, despite the well-known strategy for increasing lipid content in microalgae under nutrient restriction, it requires further specification.

Therefore, we set a task to systematise information on the use of nitrogen and phosphorus restriction to increase the lipid productivity of microalgae of different taxonomic and ecological groups to determine the directions for future research. In the course of the work, additional tasks set: (1) to study the existing relationships between nitrogen and/or phosphorus starvation and the content and productivity of lipids in microalgae, taking into account the intensity of illumination, cultivation time and the N:P ratio; (2) to analyse the variability of these dependencies at the level of strains of the same species; (3) to discuss the difficulties encountered in comparing and summarising the results available in the scientific literature, paying particular attention to terminological aspects and the methods used.

### Data collection

Data were collected from various publications containing experimental results on lipid content and lipid productivity of microalgae in batch cultures in media with varying amounts of nitrogen and phosphorus. Preference was given to those works that contained information on the species and strains of microalgae already known for their ability to synthesise lipids in large quantities and, accordingly, of commercial interest in the future. Thus, information was collected for species from various divisions and classes: Cyanobacteria, Rhodophyta, Dinophyta, Haptophyta, Cryptophyta, Heterokontophyta/Ochrophyta (Bacillariophyceae, Eustigmatophyceae, Xanthophyceae), Chlorophyta. Data on Rhodophyta and Cryptophyta were scarce. The data for the analysis were taken from tables, figures and the text of publications and summarised in the table of the accompanying material (Dataset S1). Data were recorded along with taxonomic information, culture conditions, such as light intensity, culture medium, nitrogen and phosphorus source compound, nitrogen and phosphorus concentration, growth rate and duration of the experiment. Based on the initial data, the concentrations were recalculated directly for nitrogen and phosphorus to conduct a comparative analysis of the results of studies using various sources of nitrogen and phosphorus. The N:P ratio was determined. The results of 301 experiments were included in the analysis.

To solve the tasks and to elucidate possible relationships between the content and productivity of lipids by microalgae and the amount of nitrogen and phosphorus, the method of principal components principal component analysis (PCA) was used. The study also considered the ratio N:P, the time of the experiment, and the light intensity during cultivation. Projection onto the plane of the specifics formed by PC 1 and PC 2 taking into account the vectors of variables (light intensity, nitrogen content, etc.) was indicated by dark blue and light blue areas. Calculations and plotting were carried out using the Statistica ver.12.0 software.

### Specifics of nitrogen and phosphorus used in nutritional stress

#### Nitrogen as a nutrient factor for microalgae and its main sources

Nitrogen makes up from 1 to 14% of the dry weight of microalgae [47] and provides metabolism associated with the synthesis of proteins and nucleic acids [48]. Ammonium (NH_4_+), nitrate (NO_3−_), nitrite (NO_2_−), as well as urea (NH_2_)_2_CO) can be sources of nitrogen for microalgae growth [49, 50]. Nitrogen gas is available for some cyanobacteria [51].

Evidence shows that different microalgae taxa have specific preferences regarding nitrogen sources [52]. For example, urea was the predominant nitrogen source for *Arthrospira platensis* and *Arthrospira* sp. compared to nitrates [49, 53], for *Chlamydomonas* [54] and ammonium for *Chlorella vulgaris* [50], nitrate for *Botryococcus braunii* and *Dunaliella tertiolecta* [47].

Compared to nitrates, ammonium may have an advantage as a source of nitrogen [49, 55, 56]. This is because nitrates need to be converted by nitrate reductase before they are involved in metabolism, which is costlier for cells from an energy point of view [45]. At the same time, it is known that at specific concentrations, ammonium (NH_4_+) is toxic to microalgae [57, 58]. Non-ionized ammonia, NH_3_, is even more harmful. These two forms of ammonia are linked by chemical equilibrium, but as pH and temperature increase, these shifts and the NH_3_ concentration increases [59]. There is an indication that the action of ammonium can have both short-term and long-term adverse effects on microalgae growth rate and photosynthetic activity [60]. In natural ecosystems, the conversion of NH_4_+ to nitrite (NO_2_−) and then to nitrate (NO_3−_) is a vital part of the complex nitrogen cycle, which also involves living organisms [61].

Low concentrations of nitrogenous compounds usually characterise natural (unpolluted) ecosystems. However, as a result of human activity, fluxes of nitrogen (mainly in the form of nitrate) to both fresh and coastal marine waters have increased significantly [59]. The source of a large amount of ammonium entering natural waters is urban and agricultural wastewater containing ammonium from 10 mg L^− 1^-N to 2000 mg L^− 1^-N [58]. According to Sanz-Luque et al. [62], in ocean waters, the estimated concentration of nitrate is between 7 and 31 µM; of ammonium, 0.001–0.3 µM; and nitrite, about 0.006–0.1 µM. As a rule, nitrates also predominate in surface freshwater bodies. Nevertheless, ammonium can also dominate when mineralisation processes intensify during high summer temperatures [63]. The total N concentrations of fresh surface water vary greatly. For example, in the surface waters of the forests of Northern California, USA [64], 0.03–0.1 mg L^− 1^, 0.5–2.4 mg L^− 1^ in the waters of forest and agroecosystems in China [65]; 90.0–4218.5 µg L^− 1^ in the southern alpine lakes in northern Italy [61].

The facts of changes in the composition and abundance of microalgae with changes in the trophic base of water bodies, determined primarily by the number of biogenic compounds of nitrogen and phosphorus, are well described [66, 67]. Considering that the number of nitrogen-containing compounds in natural habitats is usually low, it can be assumed that generally, microalgae are better adapted to relatively low levels of inorganic nitrogen. Accordingly, a change in its concentration, both upward and downward, may have a different effect on different types of microalgae, depending on their ecological and physiological characteristics.

#### Phosphorus as a nutrient factor

Phosphorus plays an essential role in the production of cellular components: phospholipids, nucleotides and nucleic acids [68]. The phosphorus content in the cultivation medium, like the nitrogen content, is a determining factor in the growth of microalgae [46]. At the same time, phosphorus makes up slightly less than 1% of the total biomass of microalgae, and its optimal concentration in the cultivation medium ranges from 0.001 g L^− 1^ to 0.179 g L^− 1^ [46, 69]. Phosphorus is available to microalgae as soluble phosphate (PO_4_^3−^) [68, 70]. Phosphorus restriction has been reported to increase lipid, especially TAG synthesis [68, 71]. For example, in *Isochrysis* sp. IOAC724S, when grown in a medium without phosphorus for two days, the total lipid content in the biomass increased from 21% dry weight (DW) to 55.6% DW [72]. However, phosphorus starvation can reduce lipid productivity [71]. Different microalgae species are sensitive to phosphorus deficiency, requiring additional research to understand its effect on microalgae growth and lipid accumulation [37, 68].

### N:P ratio

The stoichiometric ratio of the leading chemical elements (C:N:P) in the biomass of microalgae is one of the most critical indicators that is used to assess biochemical patterns in ecosystems, the structure of food chains, especially in conditions of increasing nitrogen and phosphorus inflow into water and soil in as a result of human activities. The optimal ratios of nitrogen and phosphorus differ for freshwater and marine, planktonic and benthic microalgae. For example, a moderate phosphorus deficiency for freshwater planktonic microalgae occurs at N:P > 22, and for freshwater benthic at N:P > 32 [73].

C:N:P ratios in habitats and organisms are closely related. Changing the balance of nutrients in the habitat changes the C:N:P stoichiometry in microalgae biomass. Accordingly, the ratio of nitrogen and phosphorus in the cultivation medium is also an important indicator that characterises their balance and makes it possible to conclude that it is optimal for microalgae growth and lipid production. These issues have already become the subject of research [37, 71, 74, 75] and suggest revealing the response of different species and taxonomic groups of microalgae to various transformations of the content nutrients in the growth medium. Species are known to have an optimal N:P ratio. However, the factors influencing the optimal N:P ratio are still poorly understood [75].

### Light intensity

Light is a determining factor in autotrophic microalgae growth and directly affects the metabolism of mixotrophic microalgae. A detailed analysis of such parameters as intensity, duration of illumination, and spectral composition of the light flux was made by us in previous work [76]. It was found that the optimal illumination intensity for the growth of microalgae, as a rule, lies in a relatively narrow range: 26–400 µmol photons m^− 2^ s^− 1^. Increasing the intensity of illumination leads to the activation of lipid synthesis. For maximum lipid productivity, different types and strains of microalgae require different levels of illumination: from 60 to 700 µmol photons m^− 2^ s^− 1^. Intense light contributes to the increase of the TAG content. Strain *Chlorella* sp. at 400 µmol photons m^− 2^ s^− 1^, had neutral lipids 3 times higher than under lighting of 40 µmol photons m^− 2^ s^− 1^, and the change in the number of membrane lipids had the opposite tendency [77]. The maximum amount of TAG in *Phaeodactylum tricornutum* was recorded at the illumination of 600 µmol photons m^− 2^ s^− 1^, and in *Tetradesmus obliquus* at the illumination of 200 µmol photons m^− 2^ s^− 1^ [77, 78].

### Time of cultivation

Microalgae growth (as an increase in cell number) has four distinct phases: lag, logarithmic (sometimes linear), stationary, and death [39, 79]. The speed of transition from one phase to another depends primarily on growth conditions, nutrient supply, the presence of stress effects, as well as the individual characteristics of taxa. The stationary phase is considered the most TAG accumulating [80, 81], which is why it is preferred in studying lipid content in microalgae. Information on lipid content in other growth phases is less common [19, 82, 83].

#### Nitrogen Starvation as a factor in stimulating lipid accumulation

Nitrogen deficiency disrupts physiological processes in microalgae. Nitrogen starvation leads to a decrease in the content of the main photosynthesis pigments [39, 84] and disruption of the functioning of photosystems (PSI, PSII). As a result, the activity of photosynthesis decreases in both eukaryotic algae and cyanobacteria [49, 85].

Cells experiencing nutritional stress activate the synthesis of reserve compounds. It is reported that a lack of nitrogen changes the direction of carbon flow from protein synthesis to carbohydrate or lipid synthesis [86]. At the same time, some types of microalgae accumulate mainly carbohydrates in nitrogen deficiency, while others accumulate lipids. For example, depletion of N from the environment leads to the suppression of *de novo* protein synthesis in *Arthrospira* and increases glycogen production and partial conversion of existing proteins into carbohydrates [49, 87]. In other cyanobacteria (*Microcystis, Synechococcus*), on the contrary, nitrogen starvation stimulates lipid accumulation [88, 89]. Recent work using the example of *Chlamydomonas reinhardtii* shows that switching metabolic pathways from lipid synthesis to carbohydrates and vice versa under nitrogen deficiency is quite complex. It is associated with competitive interactions for common carbohydrate precursors, metabolic energy, and end-product chloroplast storage space [39].

Establishing the factors that control changes in metabolic pathways and lead to a shift in biosynthesis towards lipid accumulation is very important and requires careful study. The ability to control these processes may be the key to increasing the lipid productivity of microalgae in biotechnological applications.

The consequence of stress conditions is a decrease in the growth rate of algae and, accordingly, biomass productivity. Therefore, high lipid production will depend not only on the ability of algae to synthesise them in large quantities but also on the growth rate and biomass accumulation. The best result in terms of lipid productivity will be determined by the optimal ratio of lipid content in algae cells and their growth rate.

#### Growth rate

The growth rate of microalgae is characterised in terms of absolute or relative (specific) values [90]. The most indicative are the values of the specific growth rate based on the cell densities. According to Griffiths & Harrison [91], the specific growth rate of microalgae varies over a wide range. It averages 0.96 day^− 1^ (doubling time 17 h) for cyanobacteria, for green algae – 0.69 day^− 1^ (doubling time 24 h), and in other taxa – 0.92 day^− 1^ (doubling time 18 h). The best growth rates correspond to 2.08–2.38 day^− 1^ (doubling time 7–8 h).

About half of the studies included in our analysis are accompanied by information on the growth rate of algae (Dataset S1). Available data are in the range of 0.06–5.2 day^− 1^. The group of leaders is formed by: *Micractinium* sp. ME05 (5.2 day^− 1^), *Hindakia tetrachotoma* ME03 (4.6 day^− 1^), *Scenedesmus* sp. ME02 (3.5 day^− 1^) [92], *Ettlia oleoabundans* (1.69 day^− 1^) [93], *Phaeodactylum tricornutum* CCMP 2561 (1.63 day^− 1^), *Cryptomonas baltica* NIVA-5/91(1.4 day^− 1^) [94], *Chlorella vulgaris* (1.34 day^− 1^), *Tetraselmis suecica* (1.32 day^− 1^), *Isochrysis* sp. (1.32 day^− 1^) [93], *Arthrospira platensis* (1.28 day^− 1^) [95]. There are few among those listed algae with lipid content of more than 30–40% DW. These are, for example, *Ettlia oleoabundans* and *Cryptomonas baltica*. For species accumulating lipids over 50% DW, finding information on the growth rate was not always possible. Available data suggest that it is significantly lower than species with lower lipid content. In the analysed experiments, the growth rate of such species was up to 0.62 day^− 1^. This is expected since lipid accumulation is caused by stress conditions inhibiting algae growth. Similar conclusions were also made in other works [39, 71, 72].

A decrease in the growth rate of microalgae can also occur under optimal light and temperature conditions, as well as a sufficient amount of nutrients in the environment. This may be due to an increase in cell volume, accompanied by a decrease in the intracellular transport of nutrients and a decrease in the specific content of chlorophyll *a* in cells [96]. It is also indicated that large cells have a high nitrogen uptake capacity and a large storage capacity according to their needs. Still, their growth is limited by converting nutrients into biomass. Small species show similar specific volume V (max N) compared to their larger counterparts but have higher nitrogen requirements [96]. These dependencies are traced mainly to species within the same taxonomic group [90]. Thus, a low nitrogen concentration can lead to a slowdown in the growth of microalgae. For example, George et al. [97] reported that the microalgae *Ankistrodesmus falcatus* showed slow growth in the BBM medium due to low nitrogen concentration (0.041 g L^− 1^ NO_3_-N), while BG-11 medium, where there was more nitrogen content (0.247 g L^− 1^ NO_3_-N) contributed to growth.

In periodic cultures, with an increase in nutrient deficiency, including nitrogen, a decrease in the growth rate of microalgae is also observed. In experiments with initially created conditions of low nitrogen content, this trend persists, but the decrease in growth rate occurs more rapidly. For example, the specific growth rate of *Isochrysis galbana* at a nitrogen concentration of 72, 144, and 288 mg L^− 1^ from the Log phase to the stationary one decreased from 1.02 to 1.23 to 0.32–0.36 day^− 1^, and at a nitrogen concentration of 0 and 36 mg L^− 1^ decreased from 0.71 to 0.13 day^− 1^ [39]. Accordingly, at the initial nitrogen concentration of 72, 144, and 288 mg L^− 1^ in the stationary phase, the growth rate decreased by 69.6% relative to the Log phase, and at nitrogen concentrations of 0 and 36 mg L^− 1^, by 81.7%. The highest (0.278 day^− 1^) specific growth rate in *Chlorella vulgaris* was observed at the overall Nitrate (0.041 g L^− 1^ NO_3_-N) [68]. An increase in the content of (NO_3_-N) in the cultivation medium to 0.412–0.417 g L^− 1^ reduced the rate to 0.259–0.232 day^− 1^. The restriction of nitrogen content to 0.012 (NO_3_-N) g L^− 1^ was accompanied by an even more pronounced decrease in the specific rate (0.051 day^− 1^). With the complete exclusion of nitrogen from the cultivation medium, the specific growth rate of *Chlorella vulgaris* acquired a negative value (-0.015 day^− 1^) [68]. Similar results were obtained by Kamyab et al. [98] for *Chlorella sorokiniana*: high and low nitrogen concentrations (11.0 M and 0.3–1.5 M NaNO_3_) were accompanied by low specific growth rates (0.003 and 0.006–0.022 day^− 1^, respectively), and the highest values (0.175 and 0.128 day^− 1^) corresponded to concentrations of 3.0 and 6.0 M NaNO_3_.

These results again confirm the feasibility of an individual approach to determining the required amount of nitrogen in the cultivation medium to achieve a high growth rate and high microalgae biomass or lipid productivity.

### Effect of nitrogen and phosphorus Starvation on the content and productivity of lipids in various taxonomic and ecological groups of microalgae

The content of lipids varies significantly among microalgae species of different taxonomic and ecological groups (Dataset S1). The exceptionally high content of total lipids was noted in microalgae *Prymnesium parvum* (65.79%) [99], *Vischeria vischeri* (65.16%) [100], *Nannochloropsis granulata* CCMP525 (60.35%) [101], *Chlorella vulgaris* (67.1%) [99], *Tetradesmus deserticola* JNU19 (62.4%) [102].

Over the past decades, there have been several reviews on the lipid content and lipid productivity of various microalgae species depending on the cultivation conditions. They significantly contribute to understanding these basic patterns and simplify the selection of strains for biotechnological production. An increase in lipid content in response to nitrogen restriction is considered a general pattern [36, 80]. However, according to Griffiths & Harrison [91], this pattern is not unambiguous in many cases. Some species show an increase in lipid content during nitrogen deprivation, and some offer a decrease or no change. According to their observations, Chlorophyta has a lipid content of 20–30% DW under a sufficient amount of nitrogen. With nitrogen deprivation, the lipid content changes from 18 to 64%, shifting towards an increase. For microalgae of other taxonomic groups (Cyanobacteria, Dinophyta, Haptophyta, Heterokontophyta/Ochrophyta, Chlorophyta, Euglenoza), the authors failed to identify such pronounced patterns, and the conclusion was made about their different response to nitrogen deficiency in the cultivation medium. For example, among cyanobacteria, an increase in the content of lipids with a lack of nitrogen was observed only in *Oscillatoria*. Different reactions from the point of view of lipid accumulation by microalgae in response to a decrease in the nitrogen content in the medium were also described in other works [39, 68, 98].

Using the method of principal components, we studied the relationship between the accumulation of lipids and lipid productivity of microalgae species of the main taxonomic (Cyanobacteria, Heterokontophyta/Ochrophyta, Chlorophyta, Dinophyta, Haptophyta) and ecological groups (marine and freshwater in conjunction with terrestrial ones) and the amount of nitrogen in the cultivation medium, taking into account some other cultivation conditions: light intensity, cultivation duration, phosphorus content, N:P ratio. For this analysis, an additional data table was formed from the full array of collected results containing all the necessary information on the main taxonomic and ecological groups (Dataset S1). The results, which did not have complete information on the amount and productivity of lipids, the content of nitrogen and phosphorus in the cultivation medium, the light intensity, and the duration of the experiment, were not used in XRD calculations. The result should identify hidden factors that determine these relationships and their visualisation for the studied sample of microalgae species and strains for subsequent procedures for their study and use in biotechnological practices.

### Cyanobacteria

Applying the principal component method for Cyanobacteria made it possible to identify four main factors explaining 86.30% of the total variance and obtain values that reflect the relationship between new aspects and the studied variables (Table 1). The results indicate the differences among Cyanobacteria species in terms of the effect of nitrogen nutrition on the content and productivity of lipids. As can be seen from Table 1, principal component 1 (PC 1), which accounts for 37.38% of the total variance, closely correlates with lipid productivity, nitrogen and phosphorus content, and the N:P ratio. Attention is drawn to the negative relationship between lipid productivity and nitrogen and phosphorus content and the positive relationship between lipid productivity and the N:P ratio. Thus, optimal N:P ratio and dependence on sufficient phosphorus are significant for the high lipid productivity of Cyanobacteria. PC 2 is most associated with lipid content. The weak correlation of PC 2 with other variables (except for a noticeable relationship with phosphorus content in the culture medium) suggests that the lipid content in Cyanobacteria depends on the species. PC 3 and PC 4 characterise the different needs of Cyanobacteria species during cultivation for light and nitrogen availability. For one group, nitrogen availability is negatively related to light intensity and cultivation time; for the other group, the light intensity does not act as a significant cultivation condition, and cultivation time positively correlates with nitrogen availability.

**Table 1:** Factor loading matrix of investigated variables for Cyanobacteria

Variable	PC 1	PC 2	PC 3	PC 4
Light Intensity, µmol photons m^− 2^ s^− 1^	0.06	0.44	-0.71	-0.13
Cultivation Time, day	-0.43	-0.29	-0.61	0.49
Lipid Content, % DW	-0.25	-0.81	0.10	0.04
Lipid Productivity, mg L^− 1^ day^− 1^	-0.78	0.01	0.11	-0.24
Total Nitrogen, as NO_3_-N g L^− 1^	0.50	0.19	0.52	0.38
Total Phosphorus, as PO_4_-P g L^− 1^	0.78	-0.49	-0.23	-0.26
N:P	-0.94	0.02	0.21	-0.09
Total variance, %	37.38	20.31	18.22	10.41

**Fig. 1:**
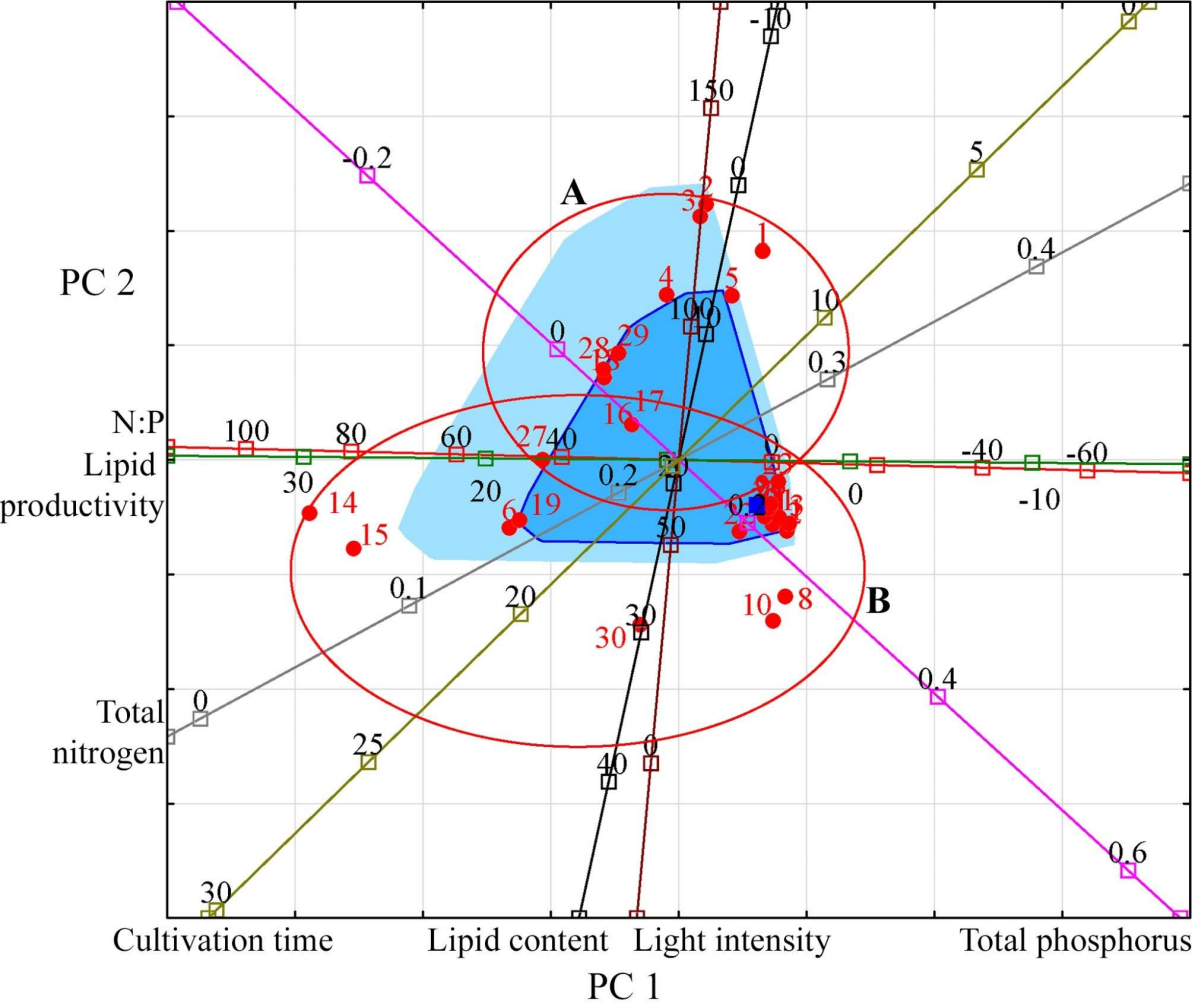
Projection of the studied variables and observations onto the factorial plane PC 1 and PC 2 for Cyanobacteria. **Indicators (variables)**: light intensity (µmol photons m^− 2^ s^− 1^); cultivation time (days); lipid content (% DW); lipid productivity (mg L^− 1^ day^− 1^); total nitrogen (g L^− 1^); total phosphorus (g L^− 1^); N:P (unit). **Observations**: species or strain numbers (circles): *Arthrospira platensis* (1–4); *Arthrospira platensis* NIOF17/003 (5); *Microcystis aeruginosa* (6, 16, 17); *Microcystis aeruginosa* CACIAM08 (7–13); *Microcystis aeruginosa* NPCD-1 (14, 15); *Oscillatoria* sp. PBGA3 (18); *Synechococcus elongatus* PCC7942 (19); *Synechocystis* sp. CACIAM05 (20–26); *Synechocystis* sp. MH01 (27); *Tolypothrix* sp. PBGA1 (28); *Tolypothrix* sp. PBGA2 (29); *Trichormus* sp. CENA77 (30). Group A: species and strains representing filamentous Cyanobacteria (*Arthrospira*, *Oscillatoria*, *Tolypothrix*). Group B: species and strains representing coccoid Cyanobacteria (*Microcystis*, *Synechococcus*, *Synechocystis*). Red circles (here and further) unite species and strains of microalgae, which are characterised by general patterns (groups A, B) in the coordinate space specified by PC 1 and PC 2

For a more meaningful interpretation of the relationships that have arisen, the projection of the studied variables and observations onto the factorial plane PC 1 and PC 2 was made (Fig. 1).

An analysis of the location of Cyanobacteria species and strains in the projection space of the first two principal components relative to the vectors of the studied variables allows us to distinguish two relatively distinct groups. Group A consists of species and strains representing filamentous Cyanobacteria (*Arthrospira*, *Oscillatoria*, *Tolypothrix*) with a low content of lipids from 2 to 11% and their productivity in the range from 3.6 to 14.37 mg L^− 1^ day^− 1^. For the cultivation of these species, the relatively high light intensity of 100–250 µmol photons m^− 2^ s^− 1^, high nitrogen concentrations, typically 0.247–0.412 g L^− 1^, and low phosphorus concentrations of 0.09 g L^− 1^ or less are predominantly used. Group B consists of species and strains representing coccoid Cyanobacteria (*Microcystis*, *Synechococcus*, *Synechocystis*). They contain up to 44% lipids, and their ability to produce lipids reaches 46.92 mg L^− 1^ day^− 1^. Within this group, there is a strain *Trichormus* sp. CENA77 represents heterocytic Cyanobacteria. The limited information required for this group of Cyanobacteria does not yet allow an appropriate analysis.

### Heterokontophyta/Ochrophyta

As a result of calculations using the principal components method, three main factors have been identified that explain 80.4% of the dispersion of variables. The factor loading matrix (Table 2) demonstrates the relationship of new factors with the analysed variables.

**Table 2:** Factor loads matrix of studied variables for Heterokontophyta/Ochrophyta

Variable	PC 1	PC 2	PC 3
Light Intensity, µmol photons m^− 2^ s^− 1^	0.43	0.58	0.49
Cultivation Time, day	0.54	-0.48	-0.46
Lipid Content, % DW	0.76	0.36	-0.42
Lipid Productivity, mg L^− 1^ day^− 1^	0.72	0.61	-0.19
Total Nitrogen, as NO_3_-N g L^− 1^	0.64	-0.18	0.36
Total Phosphorus, as PO_4_-P g L^− 1^	-0.73	0.34	-0.33
N:P	0.58	-0.69	0.08
Total variance, %	41.14	24.82	14.45

For PC 1, the highest factor load is noted for lipid content, lipid productivity, nitrogen and phosphorus content. PC 1 reveals a positive correlation between the range of lipids and lipid productivity of Heterokontophyta/Ochrophyta and the availability of nitrogen in the environment and a negative correlation with the availability of phosphorus. PC 2 indicates a relatively strong effect of light intensity on lipid productivity. Within PC 2, the N:P ratio in the culture medium is inversely related to lipid productivity. A comparison of the values and direction of the relationship (direct or inverse) of lipid productivity factor loadings, on the one hand, and nitrogen and phosphorus content, on the other hand, for PC 1 and PC 2 summarises the heterogeneity of the species group within the Heterokontophyta/Ochrophyta in their response to availability in nitrogen and phosphorus cultivation medium. This heterogeneity is additionally reflected by PC 3. This factor distinguishes the group of Heterokontophyta/Ochrophyta species, for which lipid content and an increase in nitrogen content are negatively related.

**Fig. 2:**
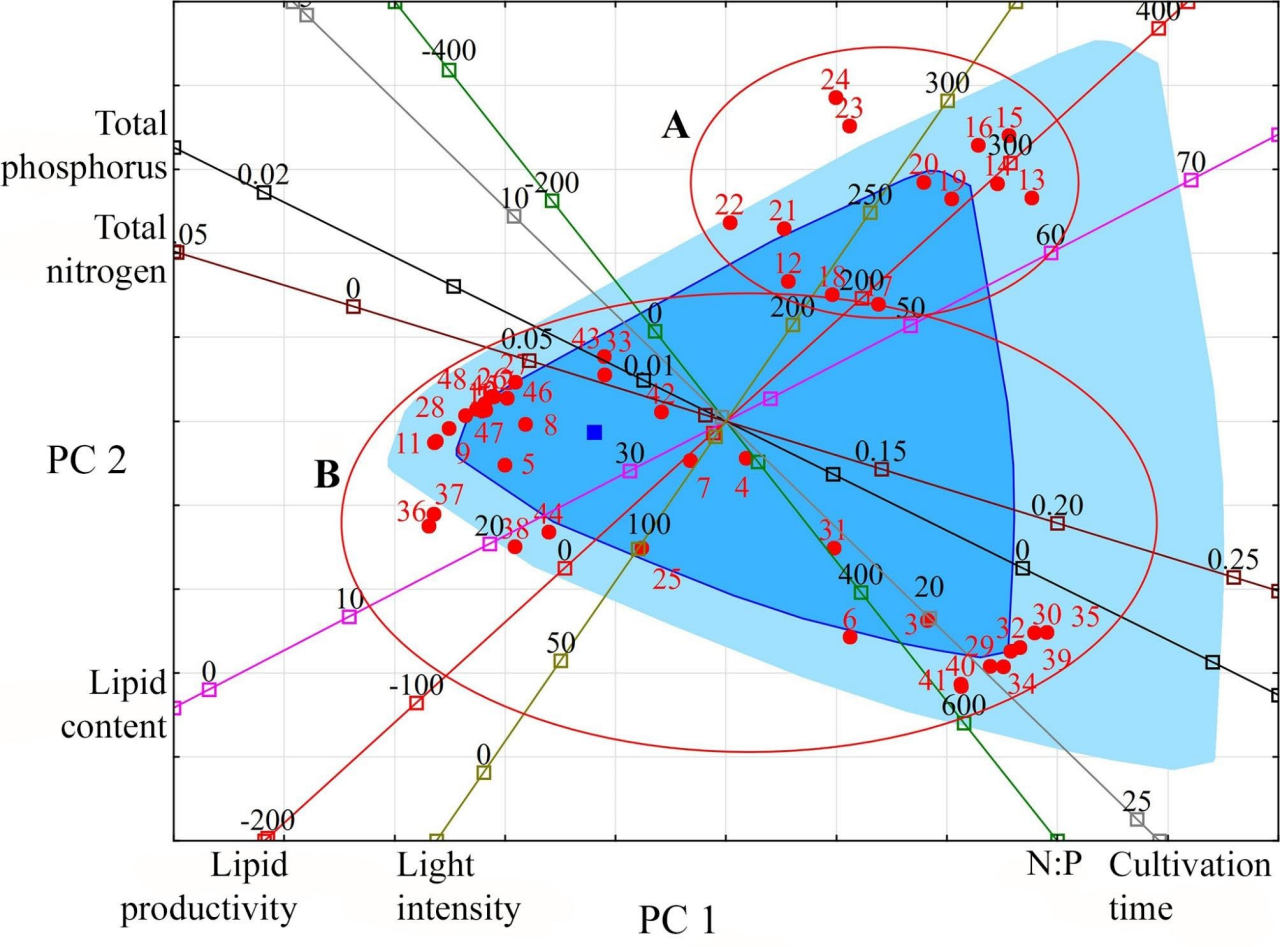
Projection of the studied variables and observations onto the factorial plane PC 1 and PC 2 for Heterokontophyta/Ochrophyta. **Indicators (variables)**: light intensity (µmol photons m^− 2^ s^− 1^); cultivation time (days); lipid content (% DW); lipid productivity (mg L^− 1^ day^− 1^); total nitrogen (g L^− 1^); total phosphorus (g L^− 1^); N:P (unit). **Observations**: species or strain numbers (circles): *Chaetoceros muelleri* (1); *Chaetoceros calcitrans* (2); *Cylindrotheca fusiformis* (3, 4); *Phaeodactylum tricornutum* (5–7); *Phaeodactylum tricornutum* CCMP 2561 (8); *Skeletonema costatum* (9); *Skeletonema* sp. (10); *Thalassiosira pseudonana* (11); *Vischeria magna* (12); *Vischeria vischeri* (13–16); *Vischeria* cf. *polyphem* (17–24); *Monodopsis subterranea* UTEX 151 (25); *Nannochloropsis* sp. F&M-M26 (26); *Nannochloropsis* sp. F&M-M28 (27); *Nannochloropsis* sp. F&M-M29 (28); *Microchloropsis gaditana* CCMP527 (29); *Nannochloropsis granulata* CCMP525 (30); *Nannochloropsis limnetica* CCMP505 (31); *Nannochloropsis oceanica* 805 (32); *Nannochloropsis oceanica* CCMP1779 (33); *Nannochloropsis oceanica* CCMP531 (34); *Nannochloropsis oceanica* IMET1 (35); *Nannochloropsis oculata* (36–38); *Nannochloropsis oculata* CCMP529 (39); *Microchloropsis salina* CCMP1176 (40); *Microchloropsis salina* CCMP537 (41); *Nannochloropsis* sp. (42–44); *Nannochloropsis* sp. CS 246 (45); *Nannochloropsis* sp. F&M-M24 (46); *Nannochloropsis* sp. F&M-M27 (47); *Ellipsoidion* sp. (48). Group A: *Vischeria* species. Group B: Eustigmatophyceae, Xanthophyceae and Bacillariophyceae species

Visualisation of the studied observations in the projection space of the first two principal components with the simultaneous plotting of vectors of various variables makes it possible to distinguish two groups of microalgae species (Fig. 2). Group A is *Vischeria* species. They are characterised by high lipid content and high lipid productivity up to 65.16% and 330 mg L^− 1^ day^− 1^, respectively (average values 52.49 ± 2.84% and 246.82 ± 23.49 mg L^− 1^ day^− 1^, respectively). Such indicators were achieved with a moderate nitrogen content of 0.138 ± 0.02 g L^− 1^, low phosphorus concentration (0.007 g L^− 1^) and high light intensity (mainly 300 µmol photons m^− 2^ s^− 1^). Group B combines the species and strains of Eustigmatophyceae (*Monodopsis*, *Microchloropsis*, *Nannochloropsis*), Xanthophyceae and Bacillariophyceae, which are located mainly along the nitrogen and phosphorus content vectors and, accordingly, differ most from each other in their response to their availability in the cultivation medium. For some *Nannochloropsis*, an increase in lipid accumulation and lipid productivity is noted with a decrease in nitrogen content (up to 0.025 g L^− 1^); others with an increase (up to 0.165 g L^− 1^). Their cultivation is usually carried out at a light intensity of 100 µmol photons m^− 2^ s^− 1^. Bacillariophyceae species in the projection space of the first two principal components along the nitrogen and phosphorus content vectors are located in a more compact group than *Nannochloropsis*. Thus, the studied sample of Bacillariophyceae is more homogeneous in its dependence on the concentration of nitrogen and phosphorus. The lipid content in Bacillariophyceae reaches up to 39.8%, and lipid productivity is up to 55 mg L^− 1^ day^− 1^. These maxima are observed at a nitrogen content of 0.025–0.247 g L^− 1^, phosphorus 0.0003 g L^− 1^.

### Chlorophyta

Significant for describing the existing relationships between the studied were four new factors that together explained 83.93% of the variance (Table 3). PC 1 is characterised by a significant negative relationship between lipid content, nitrogen content in the cultivation medium, and an increase in its content relative to phosphorus. Lipid production, as evidenced by PC 2, significantly correlates with light intensity and lipid content, according to PC 4, on the contrary, for some Chlorophyta has a negative relationship with light intensity. PC 3 is negatively related to phosphorus content and positively related to cultivation time.

**Table 3:** Factor loading matrix of investigated variables for Chlorophyta

Variable	PC 1	PC 2	PC 3	PC 4
Light Intensity, µmol photons m^− 2^ s^− 1^	-0.14	**0.83**	-0.07	**0.35**
Cultivation Time, day	0.18	-0.49	**0.49**	0.12
Lipid Content, % DW	**0.50**	0.39	0.26	**-0.58**
Lipid Productivity, mg L^− 1^ day^− 1^	-0.09	**0.72**	0.21	-0.20
Total Nitrogen, as NO_3_-N g L^− 1^	**-0.85**	-0.14	-0.27	-0.12
Total Phosphorus, as PO_4_-P g L^− 1^	0.25	-0.07	**-0.81**	-0.25
N:P	**-0.89**	-0.11	0.30	-0.20
Total variance, %	29.35	23.62	18.92	12.04

**Fig. 3:**
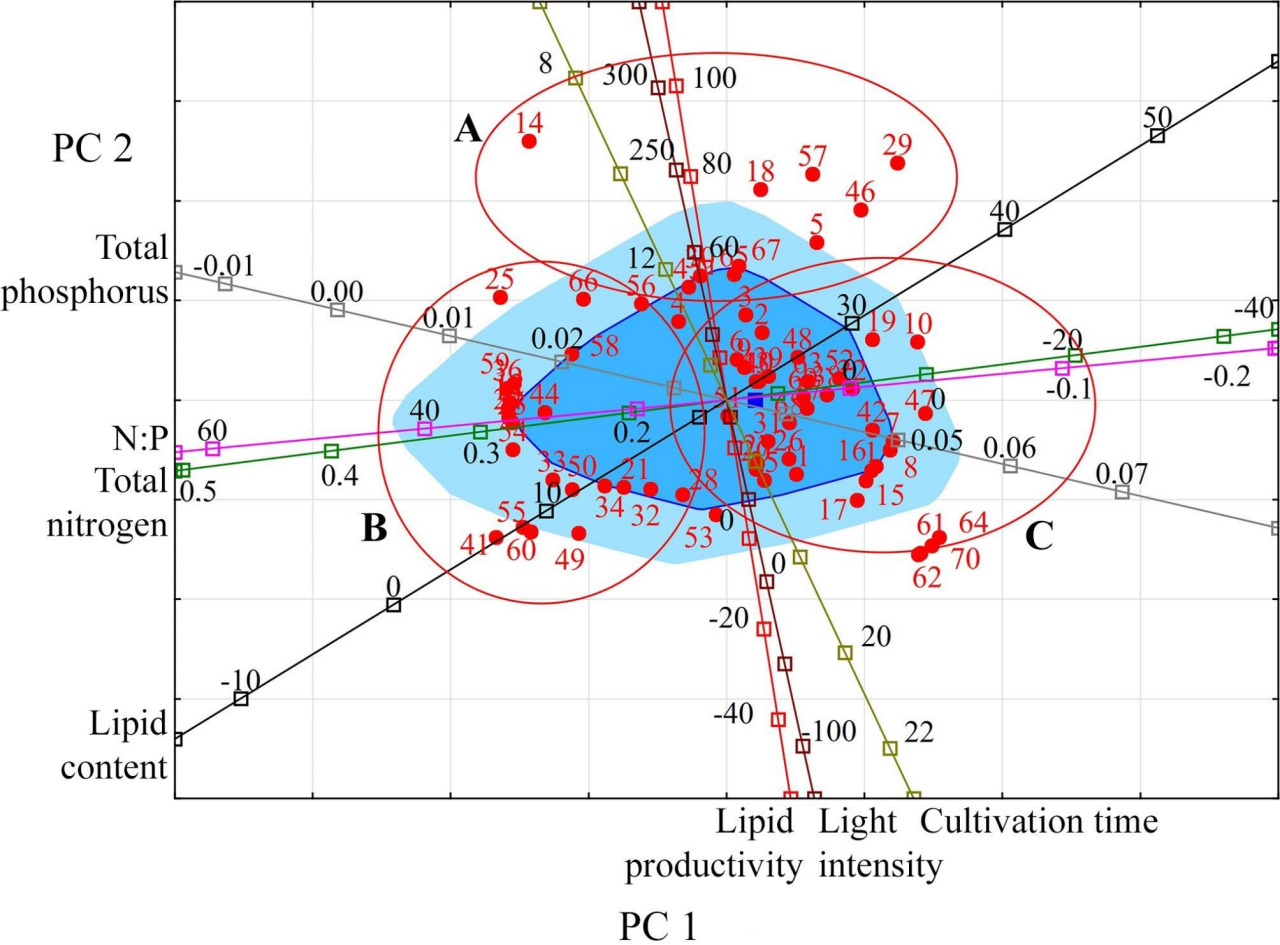
Projection of the studied variables and observations onto the factorial plane PC 1 and PC 2 for Chlorophyta. **Indicators (variables)**: light intensity (µmol photons m^− 2^ s^− 1^); cultivation time (days); lipid content (% DW); lipid productivity (mg L^− 1^ day^− 1^); total nitrogen (g L^− 1^); total phosphorus (g L^− 1^); N:P (unit). **Observations**: species or strain numbers (circles): *Tetradesmus dissociatus* (1); *Ankistrodesmus fusiformis* (2); *Ankistrodesmus falcatus* (3–5); *Chlamydocapsa bacillus* (6); *Chlamydomonadales* sp. TGA3 (7); *Chlamydomonadales* sp. TGA5 (8); *Chlamydomonas* sp. (9); *Mychonastes homosphaera* (10–11); *Chlorella sorokiniana* (12); *Chlorella* sp. (13); *Chlorella* sp. MRA-1 (14); *Chlorella* sp. PGA2 (15); *Chlorella* sp. TGA2 (16); *Chlorella* sp. TGA4 (17); *Chlorella vulgaris* (18–22); *Chlorella vulgaris* CCAP 211/11B (23); *Chlorella vulgaris* F&M-M49 (24); *Chlorella vulgaris* AG10032 (25); *Chlorella vulgaris* CCAP 211 (26–28); *Chlorella vulgaris* UTEX 395 (29, 30); *Chlorella vulgaris* CCAP 211/11B (31–35); *Chlorococcum* sp. (36); *Chromochloris zofingiensis* (37, 38); *Coelastrum microporum* (39); *Desmodesmus brasiliensis* (40); *Hindakia tetrachotoma* ME03 (41); *Hindakia tetrachotoma* PGA1 (42); *Kirchneriella lunaris* (43); *Micractinium* sp. ME05(44); *Ettlia oleoabundans* (45, 46); *Ettlia oleoabundans* REF2 (47); *Raphidocelis subcapitata* (48); *Tetradesmus obliquus* (49–53); *Desmodesmus communis* (54, 55); *Scenedesmus* sp. (56–58); *Scenedesmus* sp. DM (59); *Scenedesmus* sp. ME02 (60); *Tetraselmis chuii* (61); *Tetraselmis gracilis* (62); *Tetraselmis* sp. (63); *Tetraselmis striata* BBRR1 (64); *Tetraselmis suecica* (65–67); *Tetraselmis suecica* F&M-M33 (68); *Tetraselmis suecica* F&M-M35 (69); *Tetraselmis tetrathele* (70)

An analysis of the space formed by PC 1 and PC 2 makes it possible to distinguish three groups of Chlorophyta species (Fig. 3). Group A are fast-growing species and strains with high lipid content (up to 44–57%) and high lipid productivity (from 55 to 205 mg L^− 1^ day^− 1^) at a nitrogen content of 0.025–0.247 g L^− 1^ and light intensity up to 250 µmol photons m^− 2^ s^− 1^. These are species and strains such as *Chlorella* sp. MRA 1, *Ettlia oleoabundans*, *Scenedesmus* sp. and others. Group B combines species and strains with low lipid content and lipid productivity from 10 to 50 mg L^− 1^ day^− 1^. Such results were achieved with a nitrogen content predominantly in the range of 0.247–0.417 g L^− 1^ and a phosphorus concentration up to 0.03 g L^− 1^. These are, for example, *Chlorella vulgaris* CCAP 211/11B, *Scenedesmus* sp., *Hindakia tetrachotoma* PGA1, *Micractinium* sp. ME05 and other strains. The species grouped in group C are characterised by a high content of lipids (up to 51%). Their lipid productivity is comparable to group B species. However, it is observed at minimal amounts of nitrogen in the cultivation medium (including its complete absence) and higher phosphorus concentrations above 0.04 g L^− 1^.

### Dinophyta and Haptophyta

The relationship between the studied variables is described by three new factors (Table 4). PC 1 is characterised by a significant positive relationship with lipid content in Dinophyta and Haptophyta species and a negative relationship with nitrogen content, N:P ratio in the cultivation medium and light intensity. PC 2 shows a high negative association with the phosphorus content and cultivation time and PC 3 – with lipid productivity.

**Table 4:** Factor loading matrix of studied variables for Dinophyta and Haptophyta

Variable	PC 1	PC 2	PC 3
Light Intensity, µmol photons m^− 2^ s^− 1^	0.89	-0.02	-0.12
Cultivation Time, day	0.47	-0.83	0.13
Lipid Content, % DW	-0.75	-0.23	-0.02
Lipid Productivity, mg L^− 1^ day^− 1^	-0.12	-0.47	-0.87
Total Nitrogen, as NO_3_-N g L^− 1^	0.87	-0.16	0.07
Total Phosphorus, as PO_4_-P g L^− 1^	-0.40	-0.82	0.37
N:P	0.90	-0.10	0.02
Total variance, %	48.84	23.98	13.43

**Fig. 4:**
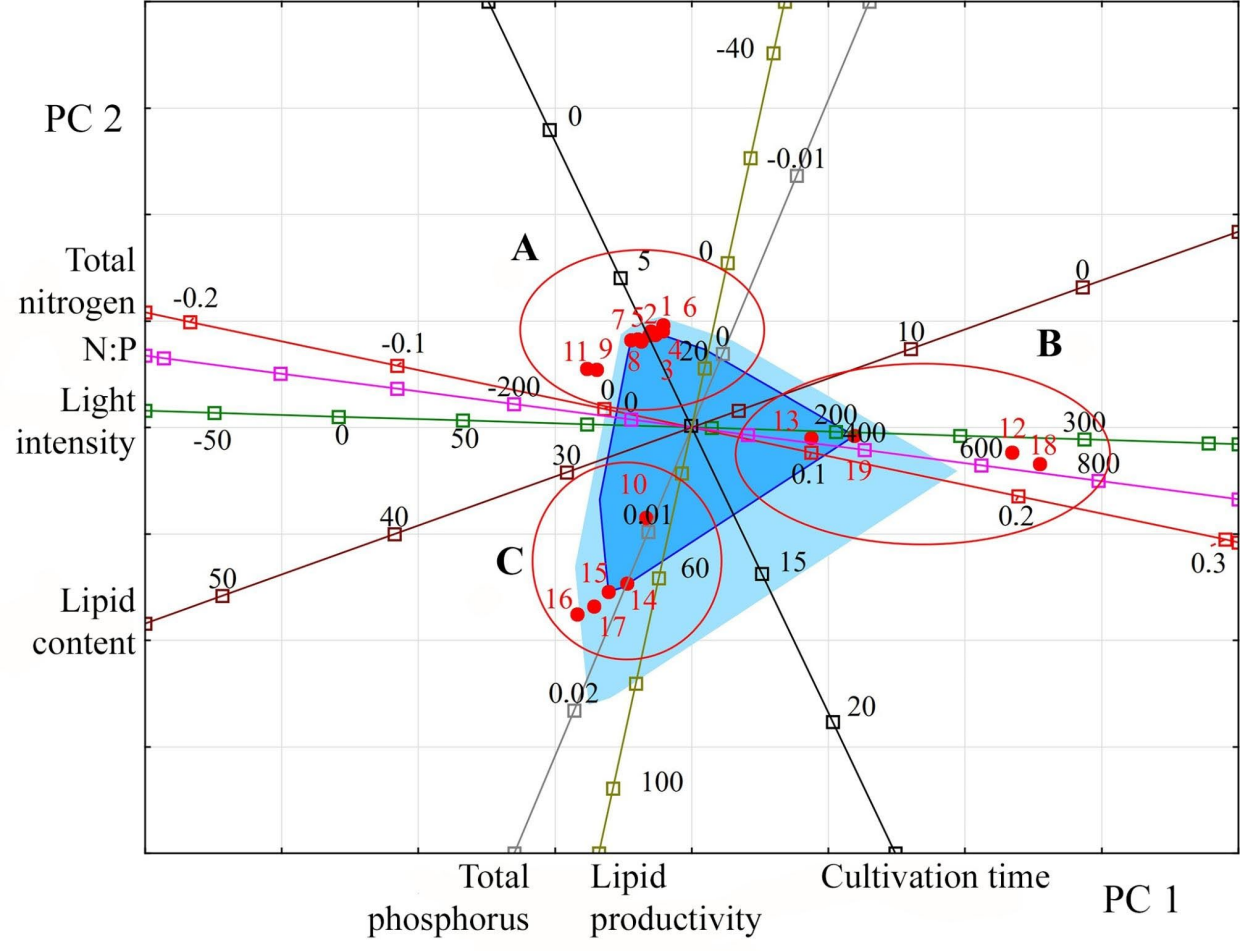
Projection of the studied variables and observations onto the factorial plane PC 1 and PC 2 for Dinophyta and Haptophyta. **Indicators (variables)**: light intensity (µmol photons m^− 2^ s^− 1^); cultivation time (days); lipid content (% DW); lipid productivity (mg L^− 1^ day^− 1^); total nitrogen (g L^− 1^); total phosphorus (g L^− 1^); N:P (unit). **Observations**: species or strain numbers (circles): *Akashiwo sanguinea* (1); *Alexandrium affine* (2); *Polykrikos geminatum* (3); *Prorocentrum minimum* strain a (4); *Prorocentrum minimum* strain b (5); *Prorocentrum triestinum* strain a (6); *Prorocentrum triestinum* strain b (7); *Scrippsiella rotunda* (8); *Isochrysis* aff. *galbana* clone T-Iso CCAP 927/14 (9); *Isochrysis galbana* (10); *Isochrysis* sp. (11–13); *Isochrysis* sp. (T-ISO) CS 177 (14); *Isochrysis* sp. F&M-M37 (15); *Diacronema lutheri* CS 182 (16); *Rebecca salina* CS 49 (17); *Pavlova* sp. (18, 19)

Within the space formed by PC 1 and PC 2, three groups of species are distinguished (Fig. 4). Group A is characterised by a lipid content in the range of 16–39%, low lipid productivity (up to 19.2 mg L^− 1^ day^− 1^) under conditions of significant nitrogen and phosphorus restriction and illumination up to 120 µmol photons m^− 2^ s^− 1^. For species from group B, there is a lower lipids content than for group A. Still, lipid productivity increases to 37.8 mg L^− 1^ day^− 1^, which occurs under nitrogen concentration conditions up to 0.247 g L^− 1^ and light intensity up to 250 µmol photons m^− 2^ s^− 1^. Species from group C are characterised by the best indicators of lipid productivity and lipid content (up to 50.2–190 mg L^− 1^ day^− 1^ and 35.5%, respectively) among the analysed samples of Dinophyta and Haptophyta species. These figures are achieved with a low nitrogen content and a high phosphorus content. At the same time, the light intensity is low. However, the cultivation time was longer than in other experiments – 14 days. From the taxonomic point of view, group A is formed mainly by Dinophyta species, while B and C are by Haptophyta.

### Marine and freshwater microalgae

When studying the lipid-producing ability of microalgae under conditions of nitrogen deprivation, the existing differences between marine and freshwater species are often noted [91, 103]. For example, mean lipid levels under nutrient-rich, nitrogen-deficient culture conditions are 22% and 36% for freshwater species and 24% and 28% for marine species, respectively [91].

To test the hypothesis that freshwater and marine microalgae react differently to the availability of nitrogen in the cultivation medium, the structure of the main components corresponding to these two ecological groups has been studied (Table 5). Terrestrial species of microalgae were considered as part of freshwater ones. Belonging to the corresponding environmental group was established based on the data provided in the analysed publications. If necessary, the data of the guides were used, where information about the typical habitats of the specific taxon was indicated.

**Table 5:** Factor loads matrix of studied variables for marine and freshwater (and terrestrial) microalgae

Variable	Marine				Freshwater and terrestrial			
	PC 1	PC 2	PC 3	РС 4	PC 1	PC 2	PC 3	РС 4
Light Intensity, µmol photons m^− 2^ s^− 1^	0.09	-0.49	0.67	-0.02	0.73	0.24	-0.12	0.16
Cultivation Time, day	-0.69	-0.14	-0.51	0.31	0.11	-0.33	0.39	-0.67
Lipid Content, % DW	-0.79	0.35	-0.08	-0.43	0.82	0.24	-0.001	-0.24
Lipid Productivity, mg L^− 1^ day^− 1^	-0.84	-0.05	0.05	-0.03	0.85	0.24	-0.27	-0.16
Total Nitrogen, as NO_3_-N g L^− 1^	-0.18	-0.86	-0.08	-0.11	-0.48	-0.06	-0.73	-0.22
Total Phosphorus, as PO_4_-P g L^− 1^	0.45	-0.57	-0.55	-0.19	-0.57	0.64	-0.04	-0.35
N:P	-0.71	-0.44	0.29	0.12	0.22	-0.85	-0.26	-0.12
Total variance, %	36.96	24.57	16.59	8.64	38.88	21.22	13.8	12.19

As can be seen from Table 5, the composition of variables that form the central relationships within each of the PCs is not the same for marine and freshwater microalgae. It is essential to emphasise the unidirectional relationship between lipid content, productivity and the N:P ratio for marine microalgae and a less pronounced but multidirectional relationship between lipid content, their productivity and the content of nitrogen and phosphorus in the composition of PC 1 in freshwater. PC 2 indicates the presence of other interactions between the range of nitrogen, phosphorus and the amount of lipids in some parts of both marine and freshwater microalgae species. For this part of marine microalgae, there is a relationship between a decrease in lipid content and an increase in the amount of nitrogen and, to a lesser extent, phosphorus in the cultivation medium. For some freshwater microalgae, the lipid content and productivity positively correlate with increased phosphorus availability. PC 3 and PC 4 account for a small part of the total variance (Table 5) and depict emerging interactions related primarily to variables such as light intensity, culture time, lipid, nitrogen and phosphorus content for a small number of microalgae species. Thus, within both marine and freshwater microalgae, there are species and strains for which the degree of availability of nitrogen and phosphorus in the cultivation medium affects lipid synthesis and lipid productivity in different ways.

**Fig. 5:**
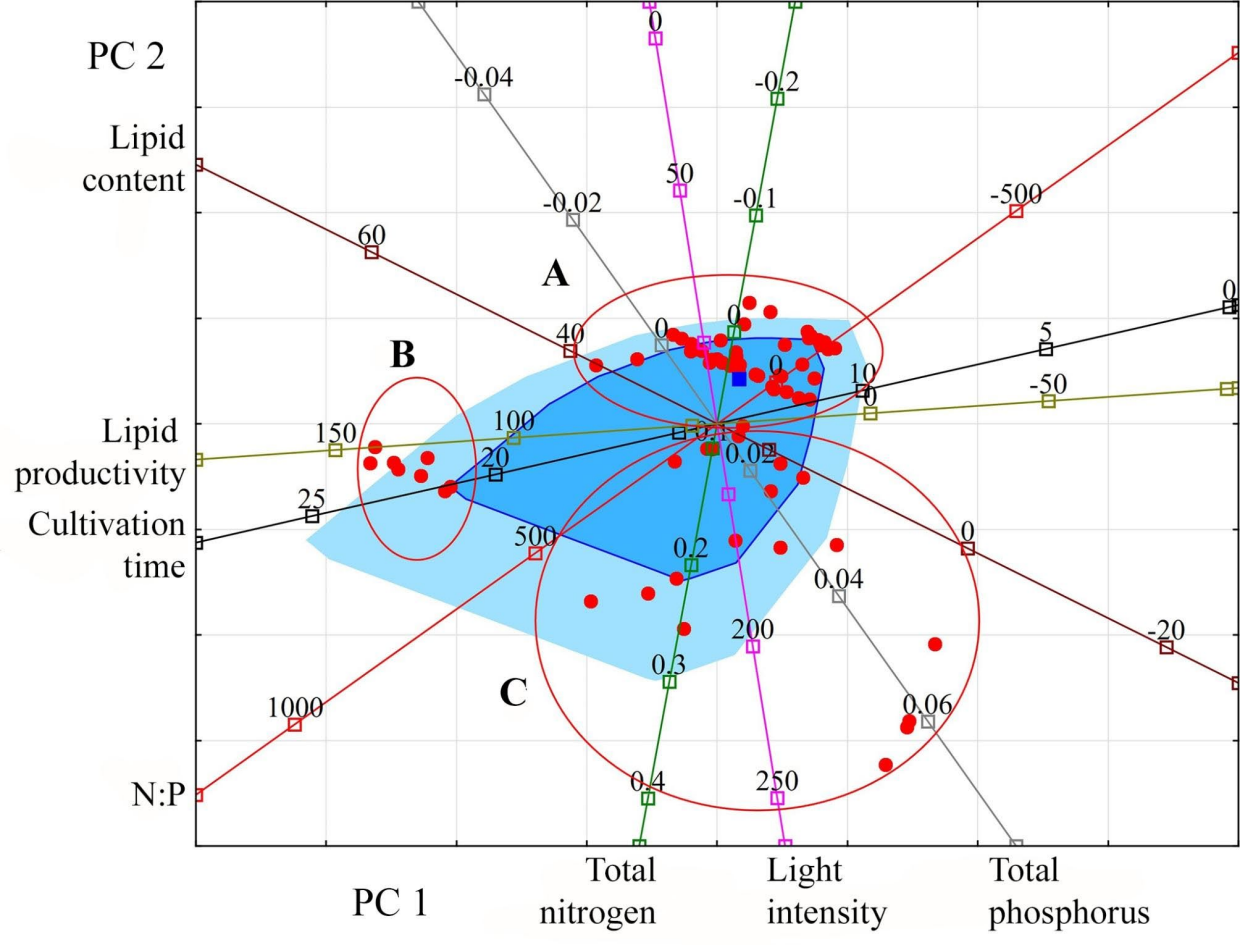
Projection of the studied variables and observations onto the factorial plane PC 1 and PC 2 for marine microalgae. **Indicators (variables)**: light intensity (µmol photons m^− 2^ s^− 1^); cultivation time (days); lipid content (% DW); lipid productivity (mg L^− 1^ day^− 1^); total nitrogen (g L^− 1^); total phosphorus (g L^− 1^); N:P (unit). Group A: *Porphyridium purpureum*; *Akashiwo sanguinea*; *Alexandrium affine*; *Polykrikos geminatum*; *Prorocentrum cordatum* (strains a, b); *Prorocentrum triestinum* (strains a, b); *Scrippsiella rotunda*; *Isochrysis* aff. *galbana* clone T-Iso CCAP 927/14; *Isochrysis galbana*; *Isochrysis* sp.; *Isochrysis* sp. (T-ISO) CS 177; *Isochrysis* sp. F&M-M37; *Diacronema lutheri* CS 182; *Rebecca salina* CS 49; *Chaetoceros muelleri*; *Chaetoceros calcitrans*; *Phaeodactylum tricornutum*; *Phaeodactylum tricornutum* CCMP 2561; *Skeletonema costatum*; *Skeletonema* sp.; *Thalassiosira pseudonana*; *Nannochloropsis* sp. F&M-M26; *Nannochloropsis* sp. F&M-M28; *Nannochloropsis* sp. F&M-M29; *Nannochloropsis oceanica* CCMP1779; *Nannochloropsis oculate*; *Nannochloropsis* sp. CS 246; *Nannochloropsis* sp. F&M-M24; *Nannochloropsis* sp. F&M-M27; *Ellipsoidion* sp.; *Tetraselmis chuii*; *Tetraselmis gracilis*; *Tetraselmis* sp.; *Tetraselmis striata* BBRR1; *Tetraselmis suecica*; *Tetraselmis suecica* F&M-M33; *Tetraselmis suecica* F&M-M35; *Tetraselmis tetrathele*. Group B: *Microchloropsis gaditana* CCMP527; *Nannochloropsis granulata* CCMP525; *Nannochloropsis oceanica* 805; *Nannochloropsis oceanica* CCMP1779; *Nannochloropsis oceanica* CCMP531; *Nannochloropsis oceanica* IMET1; *Nannochloropsis oculate*; *Nannochloropsis oculata* CCMP529; *Microchloropsis salina* CCMP1176; *Microchloropsis salina* CCMP537. Group C: *Arthrospira platensis*; *Arthrospira platensis* NIOF17/003; *Isochrysis* sp.; *Pavlova* sp.; *Cylindrotheca fusiformis*; *Phaeodactylum tricornutum*; *Nannochloropsis* sp.; *Tetraselmis suecica*

**Fig. 6:**
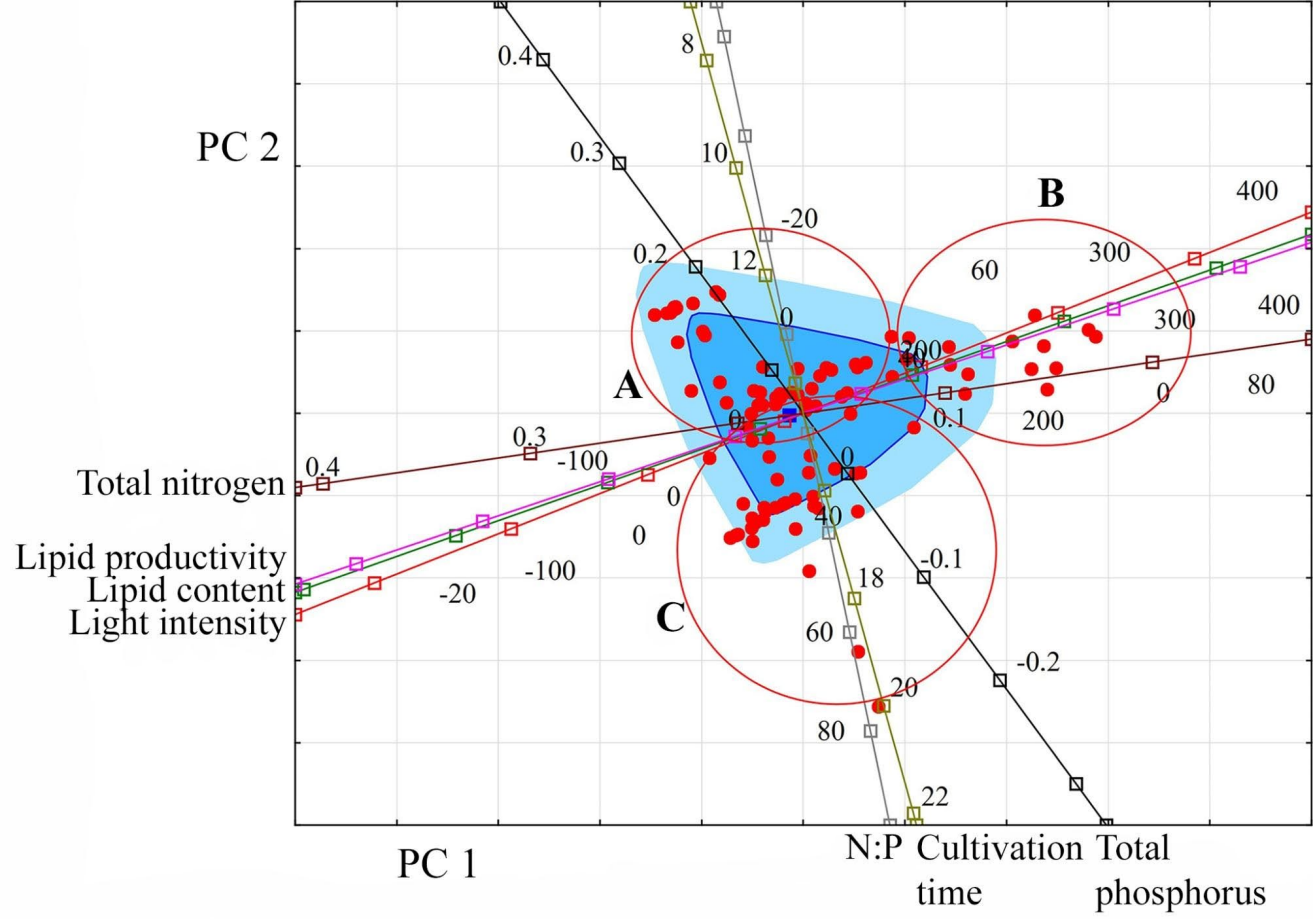
Projection of the studied variables and observations onto the factorial plane PC 1 and 2 for freshwater (and terrestrial) microalgae. **Indicators (variables)**: light intensity (µmol photons m^− 2^ s^− 1^); cultivation time (days); lipid content (% DW); lipid productivity (mg L^− 1^ day^− 1^); total nitrogen (g L^− 1^); total phosphorus (g L^− 1^); N:P (unit). Group A: *Microcystis aeruginosa* CACIAM08; *Synechocystis* sp. CACIAM05; *Trichormus* sp. CENA77; *Ankistrodesmus fusiformis*; *Ankistrodesmus falcatus*; *Chlamydocapsa bacillus*; *Chlamydomonadales* sp. TGA3; *Chlamydomonas* sp.; *Mychonastes homosphaera*; *Chlorella sorokiniana*; *Chlorella* sp. TGA2; *Chlorella* sp. TGA4; *Chlorella vulgaris*; *Chlorella vulgaris* CCAP 211/11B; *Chlorella vulgaris* CCAP 211; *Chlorella vulgaris* UTEX 395; *Chlorella vulgaris* CCAP 211/11B; *Chromochloris zofingiensis*; *Coelastrum microporum*; *Desmodesmus brasiliensis*; *Ettlia oleoabundans*; *Ettlia oleoabundans* REF2; *Hindakia tetrachotoma* PGA1; *Kirchneriella lunaris*; *Raphidocelis subcapitata*; *Scenedesmus* sp.; *Tetradesmus obliquus*. Group B: *Vischeria magna*; *Vischeria vischeri*; *Vischeria* cf. *polyphem*. Group C: *Microcystis aeruginosa*; *Microcystis aeruginosa* NPCD-1; *Oscillatoria* sp. PBGA3; *Synechococcus elongatus* PCC7942; *Synechocystis* sp. MH01; *Tolypothrix* sp. PBGA1, *Tolypothrix* sp. PBGA2; *Monodopsis subterranea* UTEX 151; *Nannochloropsis limnetica* CCMP505; *Chlorella* sp.; *Chlorella* sp. MRA-1; *Chlorella* sp. PGA2; *Chlorella vulgaris*; *Chlorella vulgaris* F&M-M49; *Chlorella vulgaris* AG10032; *Chlorococcum* sp.; *Desmodesmus communis*; *Hindakia tetrachotoma* ME03; *Micractinium* sp. ME05; *Scenedesmus* sp. DM; *Scenedesmus* sp. ME02; *Tetradesmus dissociates*; *Tetradesmus obliquus*

It is confirmed by analysing the distribution of the studied marine and freshwater algae species in PC 1 and PC 2 (Figs. 5 and 6). Three groups of species were distinguished in the composition of marine microalgae (Fig. 5). For their generalised characteristics, it is convenient to use the conditional division of the range of studied variables into three levels: “low”, “medium”, and “high”. In this case, group A species are characterised by medium lipid content, medium lipid productivity under conditions of low nitrogen and phosphorus, and low light intensity; group B – high lipid content and high lipid productivity with an average nitrogen content, a low phosphorus content and an average light intensity; group C with low lipid content, medium lipid productivity with high nitrogen and phosphorus content, and high light intensity. There is also a longer cultivation time for group B than for groups A and C when the corresponding values ​​of lipid content and lipid productivity are reached.

Among freshwater and terrestrial microalgae, three species groups were also distinguished (Fig. 6). Using a similar approach, group A is characterised by low to medium lipid content and low to medium lipid productivity under conditions of medium nitrogen, high phosphorus and medium light intensity. Group B has a high lipid content and high lipid productivity with low nitrogen, medium phosphorus, and high light intensity. Group C, with lipid content and productivity similar to group A, differs in cultivation conditions by low phosphorus content and long cultivation time.

### Effect of nitrogen Starvation on lipid content and productivity in different strains of *Chlorella vulgaris*

Analysis of the collected data, as well as the results obtained using the method of principal components, indicates that strains of the same species do not show the same pattern of changes in the content and productivity of lipids in response to changes in the nitrogen content in the cultivation medium. Therefore, we attempted to analyse the change in lipid content and their productivity using the example of *Chlorella vulgaris*, known for its ability to high lipid accumulation.

**Fig. 7:**
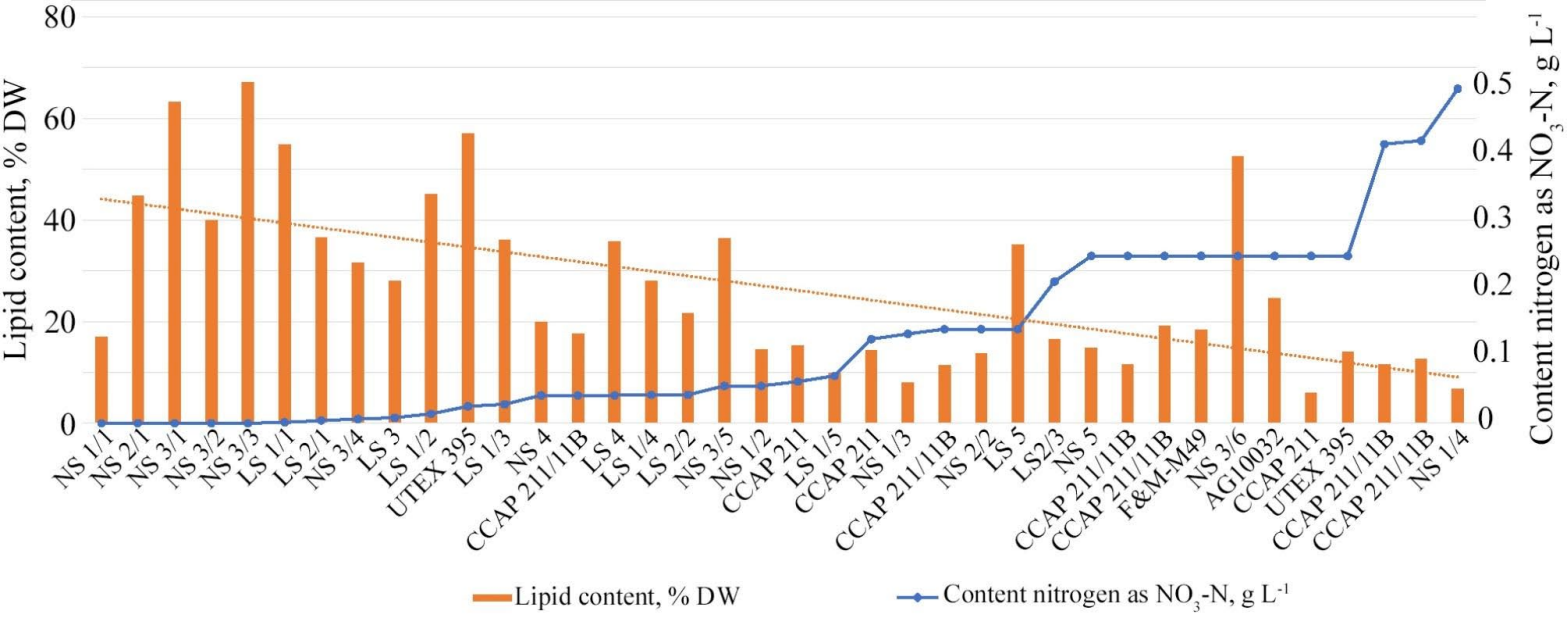
The amount of lipids in different strains of *Chlorella vulgaris* when cultivated in media with different nitrogen content. The nitrogen content was calculated as NO_3_-N g L^− 1^. For known strains, their original names were used; the rest were divided into two groups: NS, natural strains; LS, laboratory strains of personal collections. NS and LS strains from the same work have the same number. Slash numbering was used to distinguish between different experiments with these strains. List of strains and sources: NS 1/1, NS 1/2, NS 1/3, NS 1/4 [104]; NS 2/1, NS 2/2 [34]; NS 3/1, NS 3/2, NS 3/3, NS 3/4, NS 3/5, NS 3/6 [99]; LS 1/1, LS 1/2, LS 1/3, LS 71/4, LS 1/5 [105]; LS 2/1, LS 2/2, LS 2/3 [106]; LS 3 [82]; UTEX 395 [93]; NS 4 [107]; CCAP 211/11B [68]; LS 4 [108]; CCAP 211 [109]; LS 5 [110]; NS 5 [111]; CCAP 211/11B [103]; F&M-M49 [103]; AG10032 [112]

**Fig. 8:**
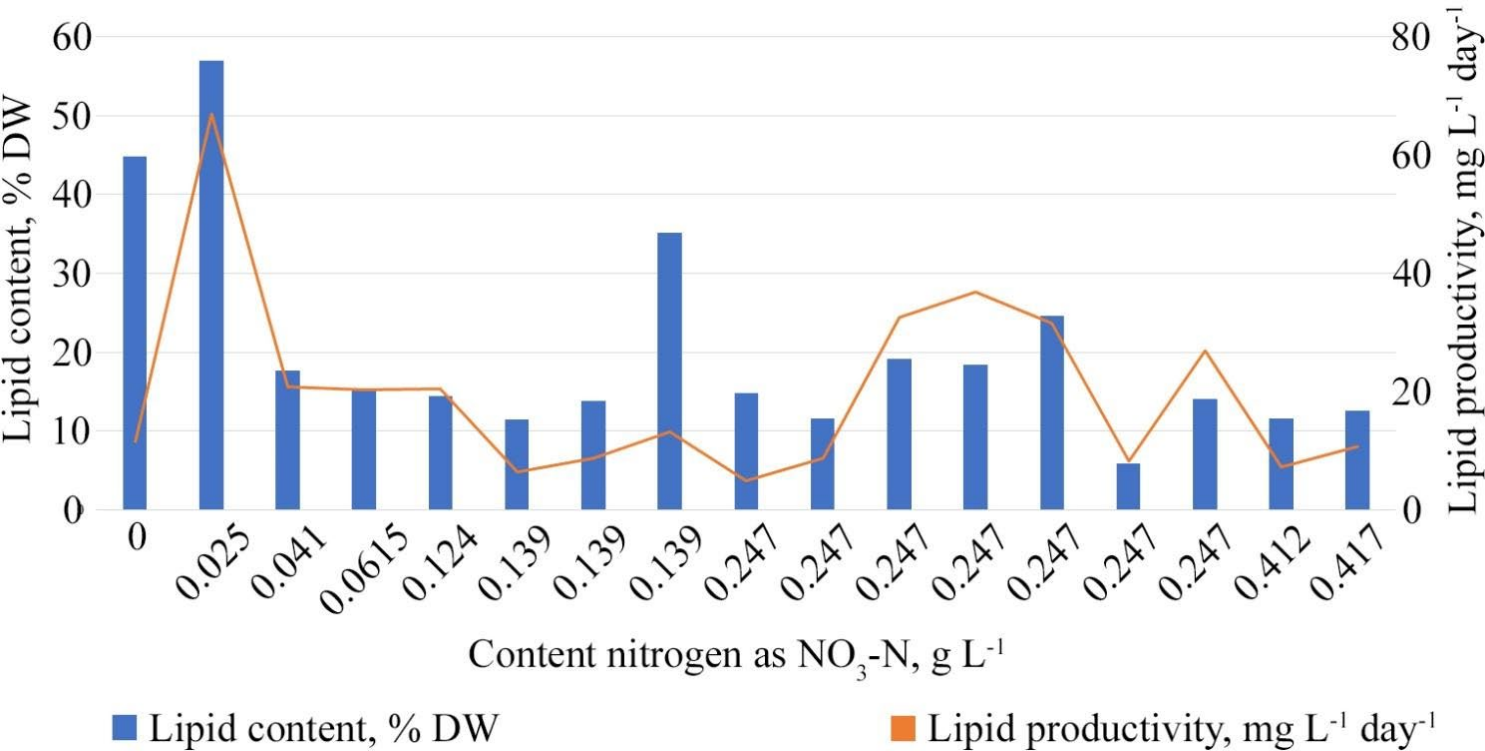
Productivity of lipids of different strains of *Chlorella vulgaris* during cultivation in media with different nitrogen content. Compiled from Converti et al. [109], Rodolfi et al. [103], Griffiths et al. [93], Mujtaba et al. [112], Abdo et al. [111], Hamedi et al. [110], Sonkar & Mallick [34], Wong et al. [68]. Nitrogen content calculated as NO_3_-N g L^− 1^

For the analysis, we used data obtained in both cultures using various cultivation media and their modifications associated with a change in nitrogen concentration (Dataset S1, lines 222–259). The sample represented a range of nitrogen concentrations from 0 g L^− 1^ to 0.494 g L^− 1^ tested in 38 experiments. The lipid content in *Chlorella vulgaris* cells in different experiments varied from 5.9 to 67.1% (Fig. 7). Lipid productivity also differed in the range of 5.0 mg L^− 1^ day^− 1^ to 204.9 mg L^− 1^ day^− 1^ except for the concentration of 0.497 g L^− 1^ for which there is no data. The constructed trend line reveals a general trend – a decrease in the content of lipids with an increase in the nitrogen concentration in the cultivation medium. However, at the same nitrogen concentration, different strains do not show lipids’ exact amount and productivity (Figs. 7 and 8).

Using the method of principal components, we found that PC 1 describes 31.83% of the total variance of factors and is positively related to the content and productivity of lipids, light intensity and negatively – to the content of nitrogen, as well as phosphorus, but a lesser extent (Table 6). PC 2 shows the maximum relationship with the N:P ratio. There is also a positive relationship between the cultivation time and nitrogen content and a negative association with the phosphorus content. PC 3 and 4 show emerging relationships between light intensity, culture time and nitrogen content. The results suggest that in *Chlorella vulgaris*, as in other Chlorophyta, the content of lipids increases with a decrease in the nitrogen content (Table 5). A specific feature of *Chlorella vulgaris* is a fairly pronounced inverse relationship between the content, the productivity of lipids and the amount of phosphorus, a significant dependence of the content and productivity of lipids on light intensity. The heterogeneity of metabolic strategies within different strains of *Chlorella vulgaris* is also evident.

**Table 6:** Factor loading matrix of the investigated variables for *Chlorella vulgaris*

Variable	PC 1	PC 2	PC 3	PC 4
Light Intensity, µmol photons m^− 2^ s^− 1^	0.73	0.18	-0.60	-0.24
Cultivation Time, day	-0.003	0.45	0.38	-0.46
Lipid Content, % DW	0.78	-0.31	-0.05	0.33
Lipid Productivity, mg L^− 1^ day^− 1^	0.59	0.02	0.15	0.12
Total Nitrogen, as NO_3_-N g L^− 1^	-0.58	0.41	-0.41	0.03
Total Phosphorus, as PO_4_-P g L^− 1^	-0.42	-0.67	-0.31	0.15
N:P	-0.06	0.90	-0.03	0.30
Total variance, %	31.83	27.02	13.82	11.81

**Fig. 9:**
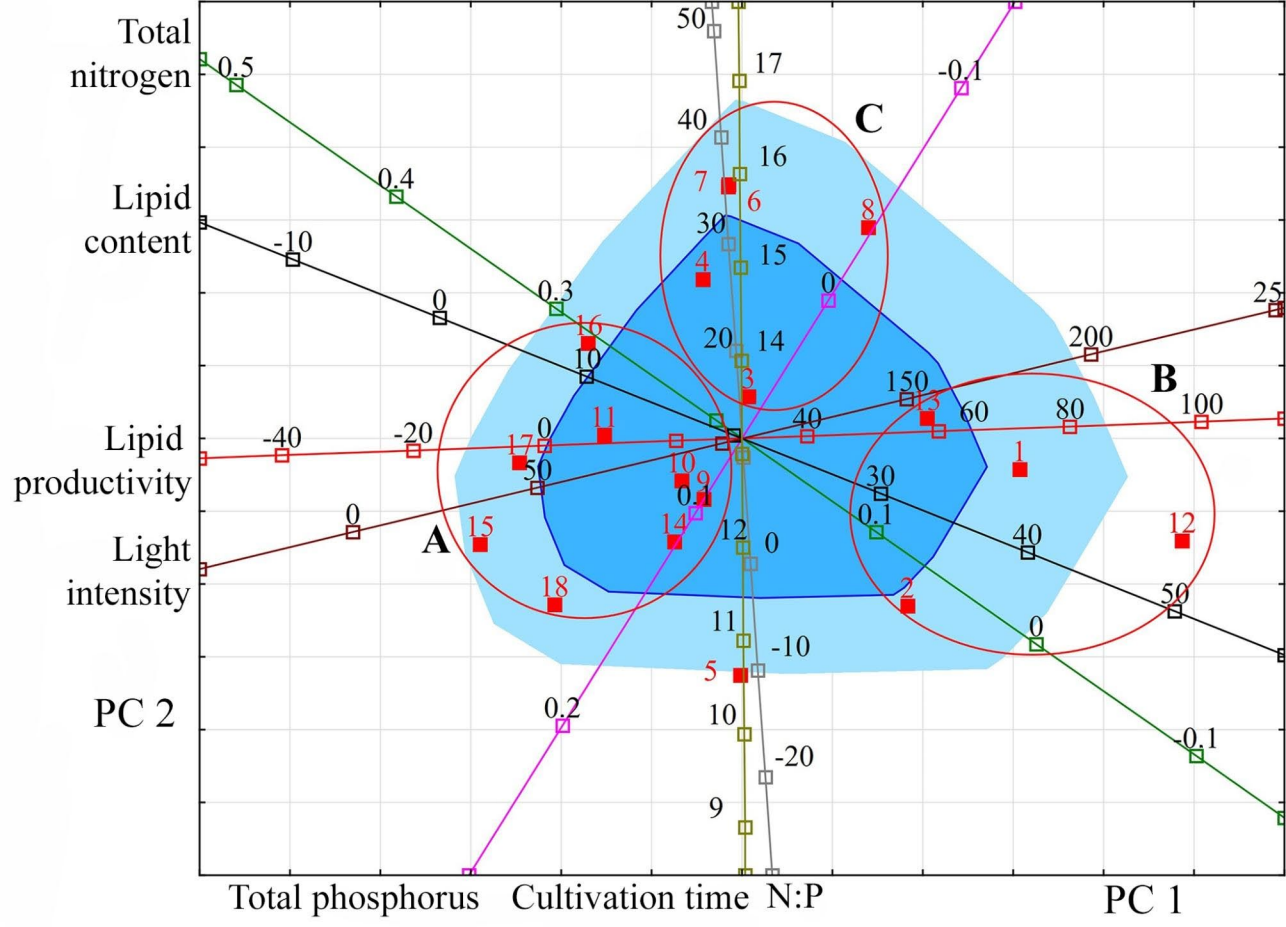
Projection of the studied variables and observations onto the factorial plane PC 1 and PC 2 for strains of *Chlorella vulgaris*. **Indicators (variables)**: light intensity (µmol photons m^− 2^ s^− 1^); cultivation time (days); lipid content (% DW); lipid productivity (mg L^− 1^ day^− 1^); total nitrogen (g L^− 1^); total phosphorus (g L^− 1^); N:P (unit). **Observations**: species or strain numbers: *Chlorella vulgaris* (5); Group A: *Chlorella vulgaris* CCAP 211 (9–11); *Chlorella vulgaris* CCAP 211/11B (14–18); Group B: *Chlorella vulgaris* (1, 2); *Chlorella vulgaris* UTEX 395 (12, 13); Group C: *Chlorella vulgaris* (3, 4); *Chlorella vulgaris* CCAP 211/11B (6); *Chlorella vulgaris* F&M-M49 (7); *Chlorella vulgaris* AG10032 (8)

This is confirmed by the analysis of the location of *Chlorella vulgaris* strains in the space of the first two principal components and their placement relative to the vectors of the studied variables (Fig. 9). Group A combined most of the experiments with the *Chlorella vulgaris* strain CCAP 211, group B with the *Chlorella vulgaris* strain UTEX 395 and several natural (wild) strains. Group C covers only natural strains and *Chlorella vulgaris* strain CCAP 211/11B grown on the BG-11 medium. A separate position is also occupied by strain number 5, which has a natural origin.

Thus, the variety of *Chlorella vulgaris* strains that are already stored in various laboratory collections or first discovered in multiple ecosystems is represented by specimens with different adaptations to the nitrogen content in the environment and different metabolic strategies to overcome stressful situations due to excess or lack of nitrogen. Differences can also be traced in the level of phosphorus availability, the N:P ratio, and adaptations to light intensity.

### Optimisation of the approach for the analysis of the amount of nitrogen and phosphorus in nutrient media

#### Nitrogen and phosphorus content in culture media

Various mineral nutrient media are used to cultivate microalgae and ensure their average growth (Table 7). Their chemical composition is designed to take into account the need for specific taxa or groups of taxa for nutrients, macro- and microelements and other specific compounds [113–118].

**Table 7:** The content of nitrogen and phosphorus in various microalgae cultivation media

Composition	Media recipe source	Basis of the media	Total nitrogen, as NO_3_-N g L^− 1^	Total phosphorus, as PO_4_-P g L^− 1^	N:P	Number of studies (296 + 5 own)
ASW (seawater)	[119]	dH2O	0.004	0.0003	13:1	11
Chu-10	[113]	dH2O	0.007	0.004	1.7:1	3
f/2	[120]	dH2O	0.012	0.001	12:1	42
Conway	[121]	dH2O	0.014	0.004	3.5:1	7
PES	[122]	Seawater	0.0152	0.0013	11.7:1	12
L1	[123]	Seawater	0.016	0.0013	12:1	10
Walne	[124]	dH2O	0.016	0.0003	53:1	15
F	[120]	dH2O	0.025	0.016	1.6:1	25
ASM-I	[114]	dH2O	0.028	0.227	0.12:1	39
BBM	[115, 116]	dH2O	0.041	0.052	0.79:1	22
Chu-13	[117]	dH2O	0.055	0.014	3.9:1	13
3N BBM	[116]	dH2O	0.124	0.052	2.4:1	14
N-8	[125]	dH2O	0.139	0.227	0.61:1	3
N 11	[34]	dH2O	0.139	0.019	7.3:1	5
BG-11	[118]	dH2O	0.247	0.007	35.3:1	66
Zarrouk	[126]	dH2O	0.412	0.089	4.6:1	7
Spirulina	[127]	dH2O	0.412	0.089	4.6:1	1
M-8	[128]	dH2O	0.417	0.227	1.8:1	1

As a source of nitrogen in nutrient media, mainly NaNO_3_ is used, less often KNO_3_ [117, 121], Ca(NO_3_)_2_ [113], Fe(NH_4_)_2_(SO_4_) [122]. Most microalgae species contain 6–9% nitrogen on a dry matter basis, so the estimated nitrogen requirement to form 1 g of biomass in 1 litter of culture medium would be about 0.07–0.08 g L^− 1^ (-N) (calculated from Zolotaryova et al. [129]). At the same time, the nitrogen content in the media, when converted to nitrogen (-N), lies in an extensive range from 0.007 g L^− 1^ to 0.417 g L^− 1^ (Table 7). For example, f/2 media [130–132] and BG-11 [83, 133, 134] differ by 20 times in the amount of nitrogen (-N). Chu-10 and M-8, used to assess the lipid content in *Chlorella vulgaris* [68, 99], differ in nitrogen (-N) by almost 60 times.

When studying the effect of nitrogen on microalgae growth, the content and productivity of lipids and the media are modified: the nitrogen concentration is reduced or increased. The maximum nitrogen content in the modified media in the analysed experiments was 0.494 g L^− 1^ [68, 104], and the minimum was 0 g L^− 1^ [39]. Thus, the range of nitrogen concentrations created during modifying cultivation media practically coincides with the range of nitrogen concentrations in unmodified media. Accordingly, data on microalgae’s lipid content and lipid productivity in some studies correlate with normal growth conditions, while in others, they correlate with stressful ones. It creates a problem in identifying the facts of stimulating the content and productivity of lipids in various microalgae and comparing the data. A comparative assessment of the ability of microalgae to accumulate lipids without specifying information on cultivation media [13, 135] can only be indicative. In this case, it is practically impossible to unambiguously exclude the effect of stimulating lipid accumulation due to low or high nitrogen concentrations in the cultivation media used and, accordingly, definitely determine the leading algae strains in terms of indicators.

The situation is similar to modifying cultivation media according to the phosphorus content. NaH_2_PO_4_, K_2_HPO_4_, and KH_2_PO_4_ are mainly used as a source of phosphorus in the media. The optimal concentration of phosphorus in the culture medium has been reported to be in the range of 0.001 g L^− 1^ to 0.179 g L^− 1^ [46, 69]. The phosphorus content lies in a more extensive range in the media used for microalgae growth: from 0.0003 g L^− 1^ to 0.227 g L^− 1^ (Table 7). This difference makes the data on the influence of the amount of available phosphorus on the growth of microalgae and changes in the content and productivity of lipids incomparable, especially without specifying the cultivation media and considering the difference in the amount of phosphorus that corresponds to them. This rule also applies to various modifications of cultural media.

As noted above, the ratio of nitrogen and phosphorus in cultivation media is considered essential for ensuring microalgae growth [68, 99]. At the same time, in cultivation media, this ratio varies and lies in the range from 0.12:1 to 53:1 (Table 7). When modifying the culture media within the limits of the experiments analysed in this work, the N:P ratio was even more significant, from 0.12:1 to 823.33:1 (auxiliary material).

Therefore, it is necessary to eliminate the existing methodological discrepancies to avoid inaccuracies in interpreting the results of studies on the effect on the growth, content and productivity of lipids by microalgae.

### Nitrogen deficiency – interpretation and main scales

The analysis of publications showed that the terms used to denote nitrogen deficiency and the amount of nitrogen corresponding to it differ in various publications. Nitrogen content is indicated as: “nitrate-starved” [34], “nitrogen-limited” [83, 93], “nitrate deficiency” [102], “nitrogen deficiency” [33], “limited nitrate feeding” [34], “strong N-limitation” [94], “moderate N-limitation” [94], “nitrogen replete” [82, 93]. In experiments with nitrogen deficiency, the culture medium can be utterly devoid of nitrogen [33] or contain some of it [102]. The amount of nitrogen, which the authors define as deficient, is not the same even in experiments with phylogenetically and/or ecologically close species or strains of the same species. For example, Li et al. [102] refer to 0.493 g L^− 1^ NaNO_3_ (equivalent to 5.8 mmol L^− 1^) for *Tetradesmus deserticola* as a concentration corresponding to deficient conditions. For *Tetradesmus* *obliquus*, a similar conclusion is drawn for significantly lower concentrations of 0.01–0.1 g L^− 1^ KNO_3_ [34]. Nitrogen deficiency for *Chlorella vulgaris*, according to various researchers, is estimated from 0.01 g L^− 1^ NaNO_3_ [34] to 0.15 g L^− 1^ KNO_3_ [93] and 0.375 g L^− 1^ NaNO_3_ [109]. It greatly complicates the subsequent use of the experimental data, especially in comparative analysis and selection of the best microalgae strains for biotechnological production.

To systematise experimental data on the relationship between nitrogen starvation and the ability of algae to synthesise lipids, Sajjadi et al. [11], Morales et al. [136] attempted to divide them into groups taking into account the N content: “N-replete” and “N-starvation”, and “N-deficiency”. However, in this case, uncertainty could not be avoided too, for example, for *Nannochloropsis* sp. “N-deficiency” is 0.025–0.105 g L^− 1^ of nitrates, for *Nannochloropsis oculata* it is 1.5–0.375 g L^− 1^, while “N-starvation” for *Nannochloropsis* sp. with 0.45 g L^− 1^ [11].

Also, to eliminate the obstacles that arise when comparing the results, in our opinion, the designation “nitrogen and phosphorus starvation” should be used only concerning a specific type of microalgae and not for the designation of nitrogen and phosphorus concentration in the cultivation medium as shown in Table 7. This approach is based on the fact that different kinds of microalgae require different amounts of nitrogen for growth. Accordingly, the same amount of nitrogen for some species will be optimal, while it will be excessive or insufficient for others. The nitrogen content in cultivation media can be divided into several ranges with the corresponding nitrogen concentration: replete -N, moderate -N, moderate N-limitation, and strong N-limitation (Table 8). Thus, it will be possible to separate the designation of the availability of the cultivation medium with nitrogen and the manifestation of the effects of nitrogen starvation or its absence, depending on the physiological and ecological characteristics of the tested microalgae species. The same is true for phosphorus (Table 8).

**Table 8:** Ranges of nitrogen and phosphorus content in microalgae cultivation media

Designation	N* g L^− 1^	Designation	Р** g L^− 1^
replete -N	˃0.4	replete -Р	˃0.2
moderate -N	0.4–0.2	moderate -Р	0.2–0.02
moderate N-limitation	0.19–0.1	moderate Р-limitation	0.019–0.01
strong N-limitation	˂0.1	strong Р-limitation	˂0.01
without nitrogen	0	without phosphorus	0

* Source of nitrogen NO_3_^−^ or NH_4_+

** Source of phosphorus РO_4_^−^

## Conclusion

1. In experiments with the content and productivity of microalgae’s lipids, various nutrient media and their modifications are used with nitrogen content from 0 to 0.494 g L^-1^ and phosphorus from 0.12 to 0.227 g L^-1^; the N:P ratio ranges from 0.12:1 to 823.33:1.
2. The range of nitrogen concentrations created by modifying the culture media coincides with the range of nitrogen concentrations in unmodified culture media. Accordingly, data on microalgae’s lipid content and lipid productivity in some studies correlate with normal growth conditions, while in others, they correlate with stressful ones. It creates a problem when summarising the facts of stimulating the content and productivity of lipids in various microalgae species and comparing the data with each other.
3. To eliminate the obstacles that arise when comparing the results, it is proposed to use the term “nitrogen starvation” only concerning to microalgal species and not for the designation of the nitrogen concentration in the cultivation medium. This approach is based on the fact that different kinds of microalgae require different amounts of nitrogen for growth. Accordingly, the same amount of nitrogen for some species will be optimal, while it will be excessive or insufficient for others. The nitrogen content in cultivation media can be divided into several ranges with the corresponding nitrogen and phosphorus concentrations: replete -N (-P), moderate -N (-P), moderate N- (P-) limitation, strong N- (P-) limitation, without nitrogen (phosphorus).
4. Changes in the content and productivity of microalgae’s lipids are associated with the amount of nitrogen and phosphorus and their ratio, light intensity and cultivation time.
5. The strength of the relationship between the content and productivity of lipids in microalgae, the amount of nitrogen and phosphorus, their ratio, light intensity and cultivation time, as evidenced by the analysis of the principal component method for microalgae of different taxonomic and ecological groups, is different and reflects the specifics of their metabolic strategies in response to change cultivation conditions.
6. With nitrogen and phosphorus deprivation, some species and strains of Cyanobacteria, Heterokontophyta/Ochrophyta, Chlorophyta, Dinophyta, and Haptophyta demonstrate an increase in the content and productivity of lipids, and some show a decrease or no changes.
7. The availability of nitrogen and phosphorus in the cultivation medium for individual species and strains of marine and freshwater microalgae affects lipid synthesis and lipid productivity in different ways.
8. Strains of the same species, as demonstrated by *Chlorella vulgaris*, are characterised by different adaptations to the nitrogen content of the environment and different metabolic strategies to cope with stressful situations due to excess or lack of nitrogen. Also, differences between strains can be traced in response to the level of phosphorus availability, N:P ratio and adaptation to light intensity.
9. Promising algae for biodiesel production may be species from Cyanobacteria, Ochrophyta, and Chlorophyta, which contain large amounts of lipids and have high lipid productivity: *Ankistrodesmus falcatus*, *Ankistrodesmus fusiformis*, *Chlorella sorokiniana*, *Chlorella vulgaris*, *Cylindrotheca fusiformis*, *Diacronema lutheri*, *Ettlia oleoabundans*, *Microchloropsis salina*, *Microcystis aeruginosa*, *Nannochloropsis granulata*, *Nannochloropsis oceanica*, *Nannochloropsis oculate*, *Phaeodactylum tricornutum*, *Prymnesium parvum*, *Rebecca salina*, *Tetradesmus deserticola*, *Tetradesmus obliquus*, *Tetraselmis suecica*, *Vischeria magna*, and *Vischeria vischeri*.

### Electronic supplementary material

Below is the link to the electronic supplementary material.

Supplementary Material 1

## Data Availability

The datasets supporting the conclusions of this article are included within the article and its additional file.
